# Allosteric Regulation of Hsp90α’s Activity by Small Molecules Targeting the Middle Domain of the Chaperone

**DOI:** 10.1016/j.isci.2020.100857

**Published:** 2020-01-21

**Authors:** Chen Zhou, Chi Zhang, Hongwen Zhu, Zhijun Liu, Haixia Su, Xianglei Zhang, Tingting Chen, Yan Zhong, Huifang Hu, Muya Xiong, Hu Zhou, Yechun Xu, Ao Zhang, Naixia Zhang

**Affiliations:** 1 Department of Analytical Chemistry, Shanghai Institute of Materia Medica, Chinese Academy of Sciences, 555 Zu Chong Zhi Road, Shanghai 201203, China; 2 CAS Key Laboratory of Receptor Research, Shanghai Institute of Materia Medica, Chinese Academy of Sciences, Shanghai 201203, China; 3 National Facility for Protein Science in Shanghai, ZhangJiang Lab, Shanghai Advanced Research Institute, Chinese Academy of Sciences, Shanghai 201210, China; 4 University of the Chinese Academy of Sciences, 19A Yuquan Road, Beijing 100049, China

**Keywords:** Molecular Biology, Molecular Structure, Cancer

## Abstract

Hsp90 is a target for anti-cancer drug development. Both the conformational events tuned by ATP/ADP and co-chaperones and the chaperoning cycle timing are required for Hsp90's fully functional display. Interfering with either one of the conformational events or the cycle timing will down-regulate Hsp90's function. In this manuscript, non-covalent allosteric modulators **(SOMCL-16-171** and **SOMCL-16-175**) targeting Hsp90α’s middle domain (Hsp90M) were developed for the first time. Multiple techniques were then applied to characterize the interactions between two active compounds and Hsp90α. Two loops and one α-helix (F349-N360, K443-E451, and D372-G387) in Hsp90M were identified responsible for the recognition of **SOMCL-16-171** and **SOMCL-16-175**. Meanwhile, the binding of **SOMCL-16-171** and **SOMCL-16-175** to Hsp90M was demonstrated to allosterically modulate the structure and function of Hsp90α’s N-terminal domain. Finally, cellular assays were conducted to evaluate the cellular activity of **SOMCL-16-175**, and the results indicate that **SOMCL-16-175** destabilizes Hsp90's client proteins and reduces cell viability.

## Highlights

• Allosteric modulators targeting Hsp90α's middle domain were developed for the first time
• Key elements in Hsp90M for the recognition of allosteric modulators were identified
• Compound SOMCL-16-175 promotes Hsp90α’s ATPase activity and reduces cell viability
• SOMCL-16-175 destabilizes Hsp90's clients without triggering heat shock response

## Graphical Abstract

**Figure:**
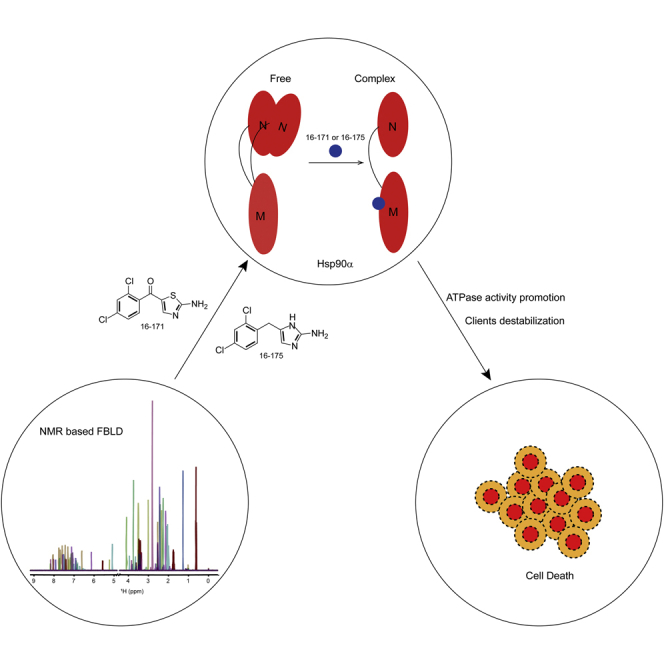

## Introduction

Protein is the main executor of life activity, and maintenance of proper protein homeostasis is essential for cell viability and growth. Protein degradation pathways (ubiquitin-proteasome system and autophagy-lysosomal pathway) and molecular chaperones, which facilitate protein folding, play central roles in maintaining protein homeostasis of living systems. Hsp90 family is a member of molecular chaperone families. And it is specified by assisting the maturation of hundreds of selected client proteins including transcription factors, steroid hormone receptors, and signaling kinases (Pearl and Prodromou, 2006, Prodromou, 2016, Prodromou, 2017, Schopf et al., 2017). By collaborating to maintain the activity of numerous proteins involved in signaling pathways and cell-cycle control, Hsp90 plays key roles in cellular signal transduction and cell growth.

In eukaryotes, such as *S. cerevisiae* and *Homo sapiens*, two cytosolic isoforms of Hsp90 are encoded and expressed: Hsc82 and Hsp82 in *S. cerevisiae* and Hsp90α and Hsp90β in *Homo sapiens*. Hsc82 and Hsp90β are constitutively expressed, whereas the levels of Hsp82 and Hsp90α are significantly up-regulated under stressful conditions, including heat shock and hypoxia (Zuehlke et al., 2015). For the human paralog Hsp90α, pathological changes including cancer can also stimulate its expression (Zuehlke et al., 2015). Owing to the involvement in cell growth and the up-regulation in multiple types of cancer, Hsp90α is becoming a promising target for anti-cancer drug development (Garg et al., 2016, Prodromou, 2009, Sidera and Patsavoudi, 2014).

Although Hsp90 homologs are expressed in a variety of organisms from bacteria to mammals, they share similar structure and function (Figure S1) (Pearl and Prodromou, 2006, Prodromou, 2016, Prodromou, 2017, Schopf et al., 2017). Hsp90 contains three well-defined structural domains: an N-terminal ATP binding domain (NTD) for ATP-binding and hydrolysis, a middle domain (MD) for client protein recognition, and a C-terminal domain (CTD) mediating Hsp90's dimerization. Hsp90 is tightly regulated by endogenous small molecules such as ATP and ADP by binding to its NTD, and co-chaperone proteins (Hop, Hsp70-Hsp90 organizing protein; p23, 23 kDa protein; Cdc37, protein encoded by the cell division cycle 37 gene; and Aha1, activator of Hsp90 ATPase 1) interacting with the subdomains of the chaperone (Ali et al., 2006, Karagoz et al., 2011, Meyer et al., 2004, Panaretou et al., 2002, Pearl and Prodromou, 2006, Prodromou, 2016, Prodromou, 2017, Schopf et al., 2017, Siligardi et al., 2002, Siligardi et al., 2004, Vaughan et al., 2008). Hsp90 possesses ATPase activity, and the binding and hydrolysis of ATP will drive conformational changes of Hsp90 associated with its different function stages: in the apo state, Hsp90 exists in a “V”-shaped conformation dimerized via its CTD; with the binding of ATP, a dimerization of Hsp90's NTD occurs, and co-chaperone proteins and client proteins are recruited; with the hydrolysis of ATP and the releasing of ADP, Hsp90 goes back to the apo “V”-shaped conformation (Pearl and Prodromou, 2000, Pearl and Prodromou, 2001, Pearl and Prodromou, 2006, Prodromou, 2012, Prodromou et al., 2000, Prodromou and Pearl, 2003). As mentioned above, tens of co-chaperones are involved in regulating the activity of Hsp90 by interacting with the chaperone protein. For example, the TPR domain (the tetratricopeptide repeat domain) of Hop could interact with the extreme C-terminal MEEVD motif (element containing conserved amino acid sequence of MEEVD) of Hsp90 and the middle domain of Cdc37 could bind to Hsp90 NTD. The binding of either of these two co-chaperones will inhibit Hsp90's ATPase activity and hinder the dimerization of the NTD of the chaperone with the presence of ATP (Roe et al., 2004, Siligardi et al., 2002, Vaughan et al., 2006, Zuehlke and Johnson, 2010). p23 is another well-studied inhibitory co-chaperone of Hsp90. It stabilizes the closed active form of Hsp90 and slows down its ATPase cycle by binding to the dimerized NTD of the chaperone (Ali et al., 2006, Karagoz et al., 2011, Martinez-Yamout et al., 2006, Zuehlke and Johnson, 2010). Among the identified co-chaperones of Hsp90, Aha1 is the only one that was reported to up-regulate the ATPase activity of the chaperone. During the chaperone cycle, Aha1's N-terminal domain binds to Hsp90's MD and induces a consequential conformation change in the MD of Hsp90. After that, the C-terminal domain of Aha1 interacts with Hsp90's NTD and promotes its ATPase activity (Meyer et al., 2004, Panaretou et al., 2002, Prodromou, 2016, Prodromou, 2017, Zuehlke and Johnson, 2010).

As mentioned earlier, the activity of Hsp90 is finely tuned by ATP/ADP and co-chaperones, and an efficient intervention of Hsp90's function could be achieved by interfering with one of the tuning steps in the working cycle of the chaperone. Multiple exogenous small molecules targeting the different structural domains of Hsp90 have been developed. They regulate Hsp90's function mainly via five ways: ATP competitive inhibitors, which block the access of ATP to the Hsp90's NTD (Prodromou, 2009, Roe et al., 1999, Sidera and Patsavoudi, 2014, Verma et al., 2016); inhibitors binding to the Hsp90's NTD and interfering with the interactions between Hsp90 and co-chaperones such as p23 and Cdc37 (Li et al., 2009, Li et al., 2018, Verma et al., 2016); compounds interacting with Hsp90's CTD and inhibiting its dimerization (Garg et al., 2016, Verma et al., 2016); allosteric activators binding to either the N-terminal domain or the interface region in between the middle domain and the C-terminal domain of Hsp90 (Bassanini et al., 2018, D'Annessa et al., 2017, Ferraro et al., 2019, Sattin et al., 2015, Yokoyama et al., 2015, Zierer et al., 2016, Zierer et al., 2014); and small molecules covalently bonding to the cysteine residue in the middle domain of Hsp90 (Li et al., 2016, Nakamoto et al., 2018, Zhang et al., 2018). Among the reported chemical compounds targeting Hsp90, ATP competitive inhibitors form a dominant group, and quite a few compounds from this group are undergoing clinical trials for the treatment of cancer (Nabi et al., 2018, Sidera and Patsavoudi, 2014, Tatokoro et al., 2015). Unfortunately, up to date, none of these inhibitors has been approved for cancer therapy. This could be at least partially attributed to the pro-survival heat shock response induced by the application of Hsp90 NTD inhibitors, which may compromise their therapeutic potential (Garg et al., 2016). A large number of inhibitors have been developed to target Hsp90 NTD, but only one non-covalent inhibitor (gambogic acid) selectively binding to Hsp90β’s middle domain has been reported (Yim et al., 2016). Meanwhile, no known non-covalent modulator targeting the middle domain of Hsp90α, which is the most pronounced target for anti-cancer drug development among Hsp90 paralogs, has been discovered yet. Client protein recognition via protein-protein interaction is the major function of Hsp90α’s middle domain (Meyer et al., 2003, Prodromou and Pearl, 2003). No binding pocket for small molecules has been experimentally identified for it. In this manuscript, the fragment-based lead compound discovery approach was applied to explore the possible binding cavity for exogenous modulators in Hsp90α’s middle domain. Two active compounds were then obtained. The findings through conducting biophysical and biochemical assays demonstrate that the active compounds could bind to the middle domain of Hsp90α and allosterically modulate the function of the chaperone. More interestingly, these allosteric modulators act as artificial activating co-chaperones of Hsp90α and accelerate its ATPase activity *in vitro*. Finally, cellular assays were subjected to test the *in vivo* activities of obtained allosteric modulators. Anti-proliferation effects and down-regulation of representative Hsp90's clients were observed in breast cancer cell lines upon the application of these compounds.

## Results

### Structural Characterization of Hsp90α’s Middle Domain

**Figure 1:**
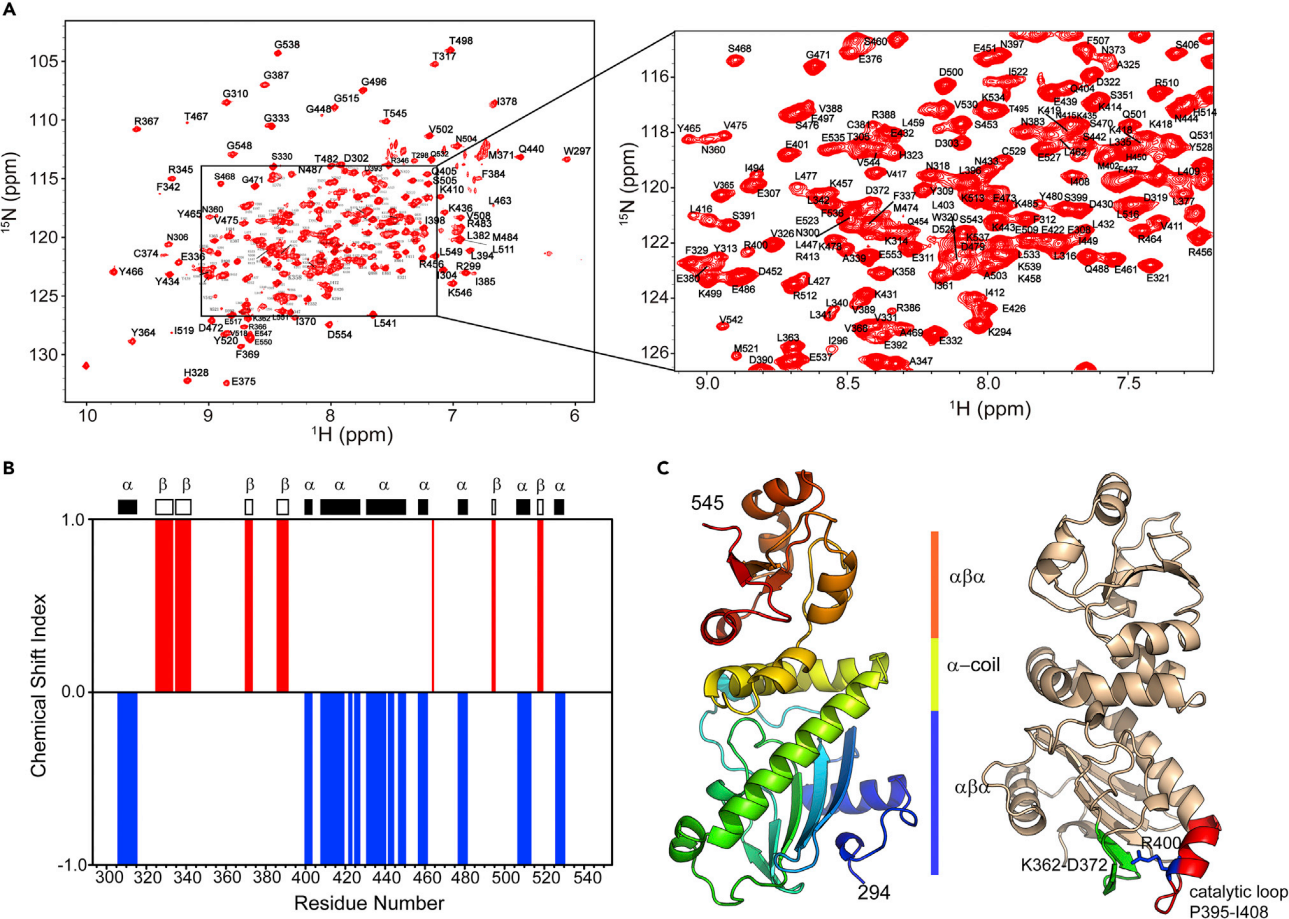
Structural Characterization of Hsp90α’s Middle Domain (A) [^1^H, ^15^N] HSQC spectra of the human Hsp90α’s middle domain in its free state. Backbone amide resonance assignments are labeled with one-letter amino acid code and sequence number. Regions with cross-peaks partially overlapped are zoomed in. (B) Chemical shift indices for the C′, Cα, and Cβ atoms of Hsp90α’s middle domain reveal its secondary structure. Identified β-strands and α-helices are presented by non-filled rectangles and filled rectangles, respectively. Unassigned residues were excluded from this analysis. (C) Ribbon representation of the crystal structure of Hsp90α’s middle domain (PDB: 6KSQ). The catalytic loop region spanning P395-I408 and its spatially adjacent fragment spanning K362-D372 are highlighted and labeled.

The good dispersion of the correlation peaks for amide nitrogen and amide proton atoms in [^1^H, ^15^N] HSQC spectrum ([^1^H, ^15^N] heteronuclear single quantum correlation spectrum) of Hsp90M indicates that it is well structured in solution (Figure 1A). The backbone resonance assignments for Hsp90M in its free state have been reported (Park et al., 2011a). These existing assignments are transferred onto our spectra and checked by 3D triple-resonance experiments including HNCA/HNCOCA, HNCO/HNCACO, and HNCACB/CACBCONH. Owing to the large size of Hsp90M, all of the 3D triple-resonance experiments were recorded by using TROSY (transverse relaxation optimized spectroscopy) scheme incorporated pulse sequences and ^15^N, ^13^C, and 70% deuterium labeled samples at 0.4 mM. Eventually, the resonances for 228 residues of a total number of 255 non-proline ones were assigned (Figure 1A). It is worth noting that the amide resonances for most of the amino acid residues in two regions including F349-K356 and K489-Y493 were entirely absent from the recorded spectra. The absence of these resonances suggests that they undergo slow conformational exchange in solution (Matsuo et al., 1999). Secondary structural elements of Hsp90M were identified by comparing the chemical shift values of CO, CA, and CB atoms with those of the corresponding residues in randomly coiled structures (Figure 1B). This analysis revealed Hsp90M to be highly structured in solution with eight α-helices and six β-strands spanning its sequence, which indicates that the global fold of Hsp90M in solution state is similar to the folding pattern revealed by its crystal structure (PDB: 6KSQ) (Figure 1C).

### Hit Generation and Medicinal Chemistry Optimization

**Figure 2:**
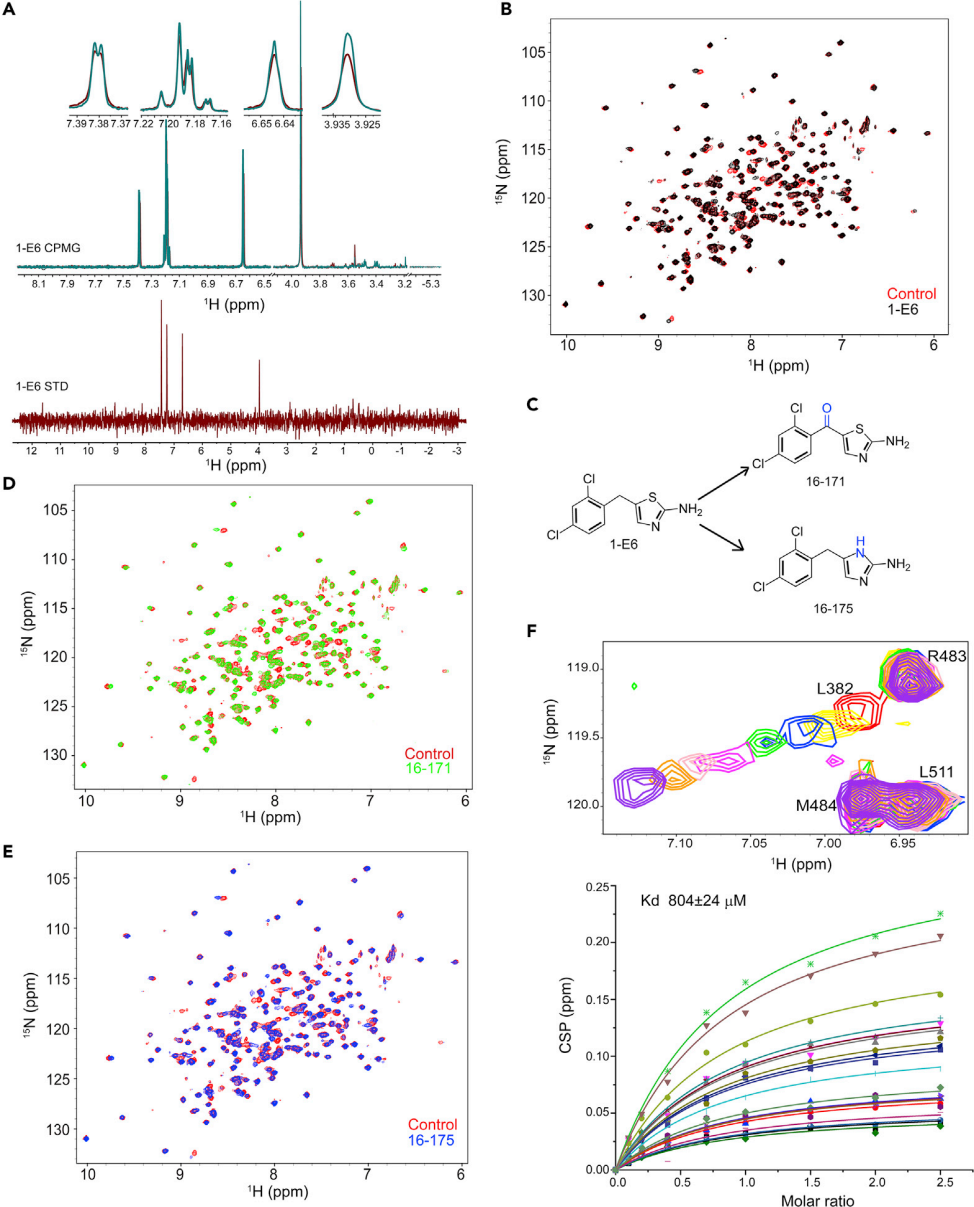
Hit Compound 1-E6 and Its Derivatives SOMCL-16-171 and SOMCL-16-175 Interact with Hsp90α’s Middle Domain (A) Ligand observed CPMG and STD spectra indicate that **1-E6** directly interacts with Hsp90α’s middle domain. (B) The specific interactions between **1-E6** and Hsp90α’s middle domain were confirmed by [^1^H, ^15^N] HSQC titration experiments. Superposition of [^1^H, ^15^N] HSQC spectra of Hsp90α’s middle domain without (red) and with **1-E6** (black, molar ratio of 1:4 Hsp90α middle domain to **1-E6**) reveals spectral changes upon hit compound binding. (C) Chemical structures of **1-E6**, **SOMCL-16-171**, and **SOMCL-16-175**. (D) The specific interactions between **SOMCL-16-171** and Hsp90α’s middle domain were demonstrated by [^1^H, ^15^N] HSQC titration experiments. Superposition of [^1^H, ^15^N] HSQC spectra of Hsp90α’s middle domain without the presence of **SOMCL-16-171** (red) and with the presence of **SOMCL-16-171** (green, molar ratio of 1:4 Hsp90α’s middle domain to **SOMCL-16-171**) reveals spectral changes upon compound binding. (E) The specific interactions between **SOMCL-16-175** and Hsp90α’s middle domain were demonstrated by [^1^H, ^15^N] HSQC titration experiments. Superposition of [^1^H, ^15^N] HSQC spectra of Hsp90α’s middle domain without the presence of **SOMCL-16-175** (red) and with the presence of **SOMCL-16-175** (blue, molar ratio of 1:4 Hsp90α’s middle domain to **SOMCL-16-175**) reveals spectral changes upon compound binding. (F) Zoomed view of the superposition of [^1^H, ^15^N] HSQC spectra of Hsp90α middle domain upon the titration of **SOMCL-16-175**. The spectra are colored according to the molar ratio of Hsp90α middle domain to **SOMCL-16-175** applied in spectrum acquisition: 1:0 (red), 1:1 (yellow), 1:2 (blue), 1:4 (green), 1:7 (magenta), 1:10 (pink), 1:15 (orange), 1:25 (purple). The dissociation constant for the binding of **SOMCL-16-175** to Hsp90α middle domain was determined by the global fitting analysis of CSP data.

In a previous work, we have setup an NMR-based platform (nuclear magnetic resonance spectroscopy-based platform) for fragment-based lead discovery, which includes a fragment library containing 539 compounds (Yu et al., 2016). Ligand-detected NMR approaches (Carr-Purcell-Meiboom-Gill relaxation dispersion NMR spectroscopy [CPMG], saturation transfer difference NMR spectroscopy [STD], and others) and target-detected NMR methods ([^1^H, ^15^N] HSQC and [^1^H, ^13^C] HSQC) are two major classes of NMR techniques that are commonly used for the NMR screening of hit compounds (Campos-Olivas, 2011). In this study, ligand-detected NMR approaches including CPMG and STD were applied in the Hsp90α middle domain-targeted hit compound screening toward the fragment library. After the primary group screening and the second cycle of single compound evaluation, one hit compound **1-E6** was identified (Figures 2A and S2). [^1^H, ^15^N] HSQC spectra of Hsp90M without or with the presence of **1-E6** confirm that the hit compound could interact with Hsp90M (Figure 2B). Since ATP competitive inhibitors targeting Hsp90's N-terminal domain compose a dominant class of exogenous molecules with the activity of modulating Hsp90's function, [^1^H, ^15^N] HSQC titration experiments were done to test if **1-E6** could interact with Hsp90's N-terminal domain. We then found, upon the addition of **1-E6**, that a minor spectral change was observed for Hsp90's N-terminal domain (Figure S3A). These data indicate that **1-E6** has a weak interaction with Hsp90's N-terminal domain. However, the interaction between **1-E6** and Hsp90's N-terminal domain shows no significant effect on **1-E6**'s binding to the middle domain of the chaperone (Figure S3B). There is no significant difference on the **1-E6**-induced spectral changes of Hsp90's middle domain without or with the presence of its N-terminal domain (Figure S3B).

Subsequently, a medicinal chemistry campaign was conducted to optimize the hit compound **1-E6** (Figure S4). First, a global manipulation of **1-E6** was initiated, including replacement of the di-chloro substitution pattern with diverse mono- or multiple halogen or non-halogen substituents, changing the methylene linker with longer alkyl or with heteroatom-containing linkers, as well as substitution of the thiazol-2-amino with alkyl or acyl groups. This round of optimization led to identification of compound **SOMCL-16-171** (Figure 2C), showing more potency and specificity against Hsp90M. Meanwhile, a further focused optimization of the thiazole with various heterocycles yielded compound **SOMCL-16-175** (Figure 2C), which showed even slightly higher potency. The binding of **SOMCL-16-171** and **SOMCL-16-175** to human Hsp90α and yeast Hsp82 (Hsp90α yeast homolog) was confirmed by ligand-detected CPMG and STD NMR data (Figures S5 and S6). Besides, compared with **1-E6**, both **SOMCL-16-171** and **SOMCL-16-175** are more potent Hsp90M modulators with enhanced CSP (chemical shift perturbation) effects upon binding (Figures 2D and 2E). The binding affinity of **SOMCL-16-175** to Hsp90M was determined to be 804 μM (Figure 2F). Meanwhile, the weak binding observed for **1-E6** to Hsp90's N-terminal domain is almost fully (**SOMCL-16-171**) or completely (**SOMCL-16-175**) abolished through compound optimization (Figure S5).

### SOMCL-16-171 and SOMCL-16-175 Are Allosteric Modulators of Hsp90α

**Figure 3:**
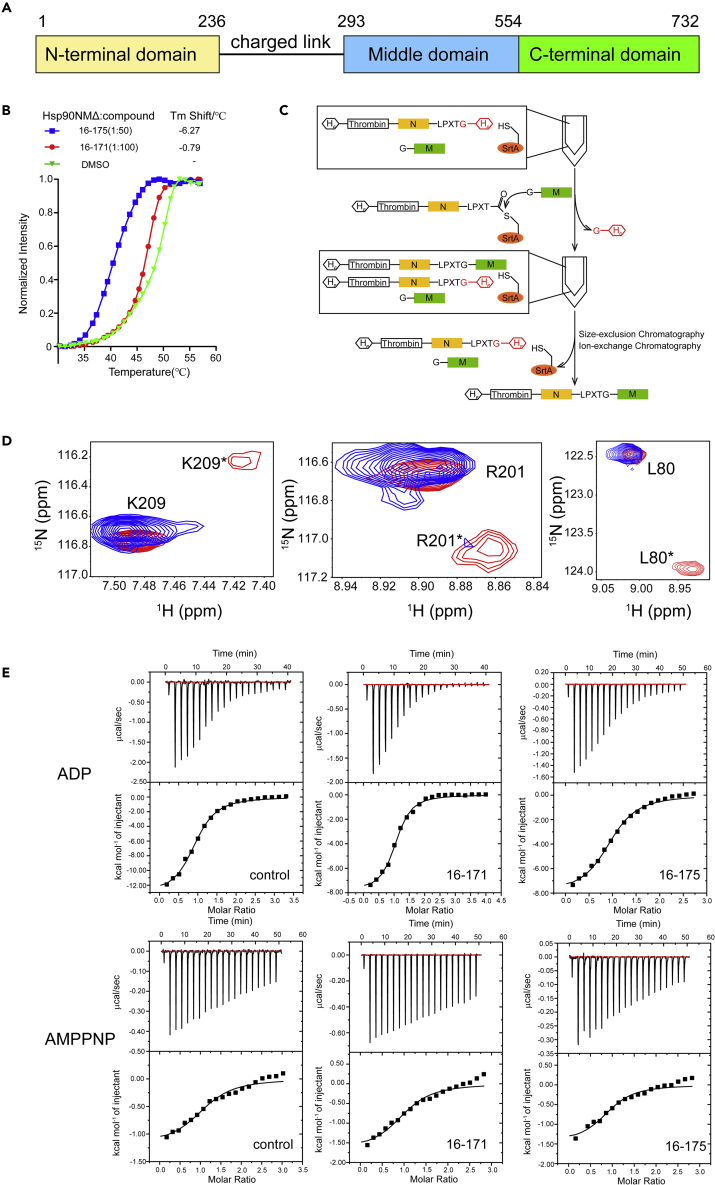
SOMCL-16-171 and SOMCL-16-175 Are Allosteric Modulators of Hsp90α (A) Schematic demonstration of Hsp90's domain architecture. (B) The shifts in *Tm* values of Hsp90NMΔ (human Hsp90α’s NTD and MD with the charged linker in between deleted) upon the binding of **SOMCL-16-171** or **SOMCL-16-175** were determined. (C) Schematic demonstration of the *in vitro* synthesis of Hsp90NMΔ with its N-terminal domain isotope labeled. (D) [^1^H, ^15^N] HSQC experiments recorded on Hsp90NMΔ with its N-terminal domain ^15^N labeled without (red) or with the presence of **SOMCL-16-171** (blue) reveal specific residues such as K209, R201, and L80 to undergo conformational shifts when the compound is present. One of the two conformations adopted by the specific residues in Hsp90's N-terminal domain is demoted by the binding of the compound. (E) Isothermal titration calorimetry experiments were applied to determine the thermodynamic parameters for the binding of ADP (upper panel) or AMPPNP (lower panel) to Hsp90NMΔ premixed with DMSO or either one of two active compounds (**SOMCL-16-171**, **SOMCL-16-175**). The fitting thermodynamic parameters are summarized in Table 1.

Hsp90 contains three defined structural domains: an N-terminal ATP binding domain (NTD), a middle domain (MD) for client protein recognition, and a C-terminal domain (CTD) (Figure 3). The ATP binding and hydrolysis in Hsp90's N-terminal domain will trigger sequential conformation changes in both Hsp90's N-terminal domain and its middle domain, and these two domains work cooperatively in the chaperone cycle of Hsp90 (Prodromou, 2012, Prodromou, 2016). Therefore, the binding of **SOMCL-16-171** and **SOMCL-16-175** to Hsp90α’s middle domain could potentially show allosteric modulation effects on the N-terminal domain of the chaperone. In this study, thermal shift assay was first applied to evaluate the global modulation effect of **SOMCL-16-171**/**SOMCL-16-175** on Hsp90α. Negative *Tm* shifts for Hsp90NMΔ (human Hsp90α’s NTD and MD with the charged linker in between deleted), Hsp90 (human Hsp90α), and Hsp82 (Hsp90α yeast homolog) were observed upon the binding of **SOMCL-16-171** and **SOMCL-16-175** (Figures 3 and S7), which indicate that the binding of the compounds could induce structural changes of Hsp90α and decrease its thermal stability. The thermal shift data suggest that **SOMCL-16-171** and **SOMCL-16-175** could potentially work as allosteric modulators of Hsp90α.

After the evaluation of the global modulation effect of two compounds on Hsp90α by thermal shift assay, the NMR method was further used to verify if the compound binding will affect the structure of Hsp90α’s N-terminal domain. To gain a clear view of the possible long-range allosteric modulation effect of the compound on Hsp90α’s N-terminal domain, a domain-specific isotope labeling approach was applied (Figure 3). Unlabeled Hsp90M and 100% ^15^N, 90% deuterium-labeled Hsp90N were expressed in *E. coli* and purified by a combination use of nickel affinity chromatography and size-exclusion chromatography. These two protein samples were then ligated together under the catalysis of the engineered Sortase A, and Hsp90NMΔ sample with its N-terminal domain selectively labeled and detected in NMR experiments was then obtained and submitted to [^1^H, ^15^N] HSQC spectrum acquisition. According to the NMR data, multiple amino acid residues including L80 and R201 in Hsp90α’s N-terminal domain adopt two conformations in solution (the resonance assignments for Hsp90α’s N-terminal domain were extracted from the reported literature [Jacobs et al., 2006, Park et al., 2011b, Zhang et al., 2015]), and two corresponding resonance peaks for each residue were observed (Figure 3). However, with the addition of active compound, one of the two resonance peaks for each amino acid residue disappeared in the recorded [^1^H, ^15^N] HSQC spectrum, which indicates that the binding of the compound allosterically shapes the structure of Hsp90α’s N-terminal domain (Figure 3).

As mentioned earlier, Hsp90's N-terminal domain has ATPase activity and the ATP binding and hydrolysis are intimately coupled to the function cycle of the chaperone. Therefore, to test if the allosteric modulation of **SOMCL-16-171** and **SOMCL-16-175** on Hsp90α’s N-terminal domain will affect its function-coupled states, ITC (isothermal titration calorimetry) experiments were carried out. We then found that in comparison with the Hsp90NMΔ in apo state, the pre-incubation of Hsp90NMΔ with **SOMCL-16-171** or **SOMCL-16-175** only presents minor effects on the binding affinities (Kd values) of ADP and ATP analog (AMPPNP) to Hsp90α’s N-terminal domain (Figure 3 and Table 1). However, the contributions of entropy (TΔS) and enthalpy (ΔH) to the binding of ADP and AMPPNP to Hsp90α are modified by the presence of the compounds (Figure 3 and Table 1). The ITC data suggest that, with the addition of the compounds, the entropy contribution (TΔS) for ADP and AMPPNP (especially for ADP) binding are significantly enhanced (Table 1). For example, when **SOMCL-16-171** and **SOMCL-16-175** were not or were pre-mixed with Hsp90NMΔ, the TΔS values for ADP:Hsp90NMΔ system were determined to be −4.84, −0.41, and −1.16 kcal·mol^−1^, respectively (Table 1). The observed gain in entropy for AMPPNP/ADP:Hsp90NMΔ systems could be attributed to the conformational changes of Hsp90NMΔ and/or the perturbation of hydration network of the chaperone protein, which are induced by the binding of **SOMCL-16-171** or **SOMCL-16-175**. Since the water/hydration network could be considered as a component of protein structure, the perturbation of hydration network caused by ligand binding is therefore intimately coupled to protein conformational changes (Biela et al., 2013, Chandler, 2005, Darby et al., 2019). Therefore, the ITC data confirm that the binding of the active compounds to Hsp90α’s middle domain does allosterically modulate the conformations of Hsp90α’s N-terminal domain in solution.

**Table 1:** Thermodynamic Parameters of the Hsp90NMΔ:ADP and Hsp90NMΔ:AMPPNP Systems Measured by ITC Experiments

Ligand	Compound	N	Kd (μM)	ΔH (10^3^ cal mol^−1^)	TΔS (10^3^ cal mol^−1^)
ADP	DMSO	0.982 ± 0.06	7.3 ± 1.0	−11.96 ± 0.4	−4.84
	16–171	1.05 ± 0.02	3.4 ± 0.045	−7.99 ± 0.18	−0.41
	16–175	0.967 ± 0.03	6.3 ± 1.2	−8.37 ± 0.32	−1.16
AMPPNP	DMSO	1.04 ± 0.08	14.1 ± 4.6	−2.27 ± 0.24	4.45
	16–171	1.01 ± 0.07	10 ± 3.7	−1.68 ± 0.16	5.24
	16–175	0.92 ± 0.07	8.7 ± 3.4	−1.46 ± 0.16	5.54

### Characterization of the Interactions between SOMCL-16-171/SOMCL-16-175 and Hsp90α’s Middle Domain

**Figure 4:**
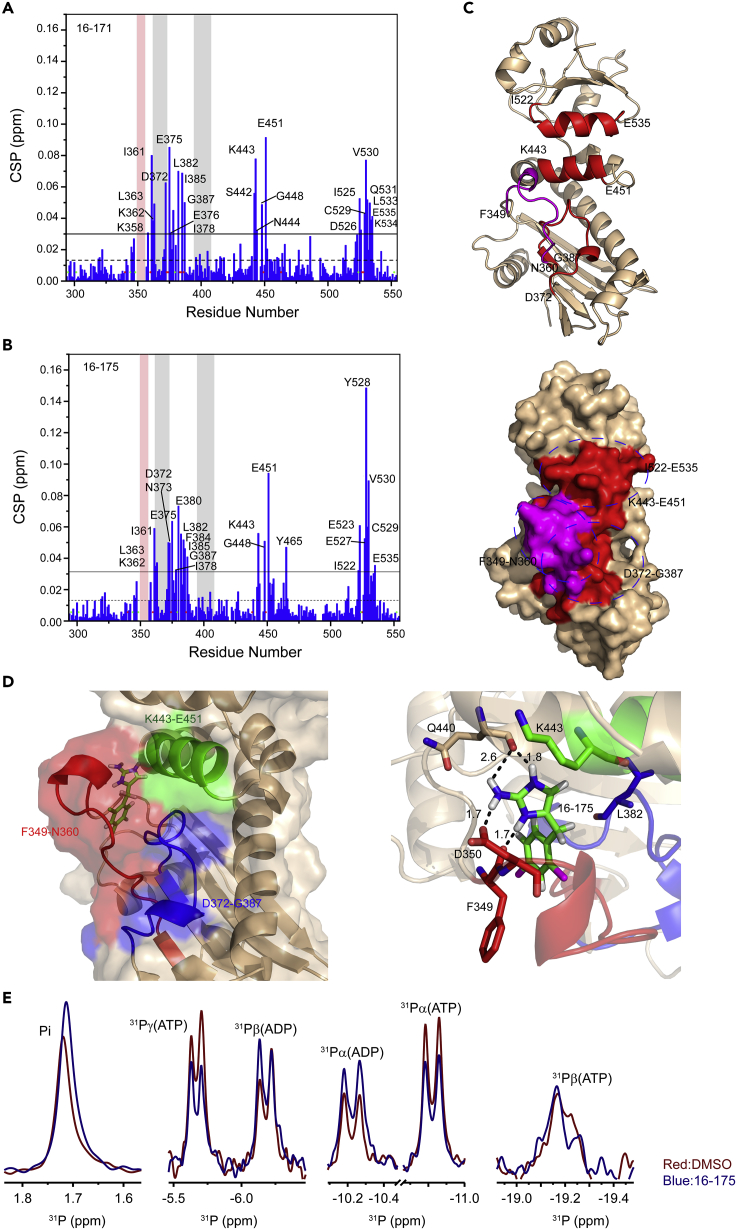
Characterization of the Interactions between SOMCL-16-171/SOMCL-16-175 and Hsp90α’s Middle Domain (A and B) Amide chemical shift perturbation analysis reveals the residues of Hsp90α’s middle domain involved in binding **SOMCL-16-171 or SOMCL-16-175**. The mean and the mean + S.D. value are indicated by dashed line and solid line, respectively. The residues with their CSPs greater than mean + S.D. are labeled. The prolines and the residues with their resonances undergoing significant attenuation upon the addition of **SOMCL-16-171 or SOMCL-16-175** are indicated with green dot and red dot, respectively. The catalytic loop (P395-I408) and the β-strands spatially close to it (K362-D372) are highlighted in gray. The fragment spanning F349-K356, which undergoes slow conformational exchange in solution, is highlighted in pink. (C) According to the CSP data shown in (A) and (B), one loop region spanning D372-G387 and two α-helices including K443-E451 and I522-E535 are identified to play key roles in the recognition of active compounds. These three regions are colored in red in both of the ribbon and the surface presentation of Hsp90M crystal structure (PDB: 6KSQ). The loop region spanning F349-N360, which undergoes conformational exchange in solution and is spatially close to D372-G387 and K443-E451, is colored in magenta. (D) Expanded view of the possible binding mode of **SOMCL-16-175** to Hsp90α’s middle domain. Carbon, nitrogen, chlorine, and hydrogen atoms of the compound are colored green, blue, magenta, and white, respectively. The distances of the hydrogen bonds to **SOMCL-16-175** are provided and indicated in black. Residues L382 and K443, which were selected for mutagenesis study, are highlighted and labeled. (E) The ^31^P NMR spectra data suggest that the application of **SOMCL-16-175** promotes the ATPase activity of Hsp82 (Hsp90α yeast homolog). The ATP hydrolysis process catalyzed by Hsp82 was monitored by acquiring 1D ^31^P spectra. Superposition of 1D ^31^P spectra of Hsp82:ATP (3 μM:1 mM) reaction system without (red) and with the presence of **SOMCL-16-175** (500 μM, blue) acquired at the time point of 3 h after the initiation of the reaction.

To map the interacting sites of **SOMCL-16-171** and **SOMCL-16-175** in Hsp90α’s middle domain, [^1^H, ^15^N] HSQC NMR titration experiments were performed (Figure 2). The interactions between Hsp90α’s middle domain and two compounds are revealed by the CSP analysis data extracted from the [^1^H, ^15^N] HSQC spectra (Figures 4A and 4B). The residues with their chemical shifts perturbed and attenuated significantly upon the addition of **SOMCL-16-171** and **SOMCL-16-175** in Hsp90α’s middle domain are identified as follows: L340, K358, N360, I361, K362, L363, D372, N373, C374, E375, E376, I378, L382, N383, F384, I385, R386, G387, S442, K443, N444, G448, I449, E451, I525, D526, E527, Y528, C529, V530, Q531, L533, K534, E535 for **SOMCL-16-171** and L340, N360, I361, K362, L363, D372, N373, C374, E375, I378, E380, L382, F384, I385, R386, G387, Y438, K443, G448, I449, E451, Y465, I522, E523, I525, E527, Y528, C529, V530, K534, E535 for **SOMCL-16-175** (Figures 4A and 4B). The perturbed residues in Hsp90α upon the binding of **SOMCL-16-171** and **SOMCL-16-175** both localize to four fragments spanning N360-L363, D372-G387, K443-E451, and I522-E535, which are spatially close to each other (Figure 4C). And this suggests that these four regions modulate the binding of the compounds to Hsp90M. According to the backbone resonance assignments data, the region spanning F349-K356 of Hsp90M undergoes slow conformational exchange in solution and the resonances of the residues in this region are fully absent from the recorded NMR spectra. The slow conformational exchange in this gate-like fragment is expected to favor the recognition of the active compounds by Hsp90α’s middle domain. The conclusion achieved by CSP analysis was further confirmed by the mutagenesis study results. Compared with the binding of **SOMCL-16-175** to wild-type Hsp90M, its interactions with Hsp90M F349A and Hsp90M D350A mutants are almost fully abolished, and its interactions with Hsp90M L382A and Hsp90M K443E mutants induce less significant CSPs of the corresponding residues (Figure S8). Meanwhile, the binding of **SOMCL-16-175** to HSP90M Y528A mutant and its interaction with wild-type Hsp90M induce comparable CSPs of the representative residues (Figure S8). The mutagenesis study data suggest that the α-helix spanning I522-E535 might not be involved in the direct binding of **SOMCL-16-175** and the compound binding cavity in Hsp90M is composed of F349-N360, D372-G387, and K443-E451. The observed significant CSPs in the helical region spanning I522-E535 are most possibly from the conformational changes induced by the compound binding. To further define the binding cavity for **SOMCL-16-175** and reveal the possible binding pose of **SOMCL-16-175** in Hsp90M, molecular docking approach was applied. The docking grid was centered on the centroid of eight residues: Phe349, Leu363, Asp372, Gly387, Lys 443, Glu451, Ile522, and Glu535, which were chosen according to the CSP analysis data. The docking model was then obtained, and the binding pocket composed of F349-N360, D372-G387, and K443-E451 for **SOMCL-16-175** in Hsp90M was confirmed (Figure 4D). According to the published literature, the binding pocket for exogenous small molecules in Hsp90α’s middle domain identified by us is also found in Hsp90β’s middle domain (Yim et al., 2016).

After the identification of the binding sites of two compounds in Hsp90M, we then tested if the compound binding would allosterically modulate the ATPase activity mainly exerting by Hsp90α’s N-terminal domain. As it has been reported, two fragments including K362-D372 and P395-I408 in Hsp90M are involved in the promotion of Hsp90's ATPase activity (Meyer et al., 2003, Prodromou, 2016). P395-I408 is named as the catalytic loop, which promotes the ATP hydrolysis process by moving to an open active state and interacting with the γ-phosphate of ATP through the conserved arginine residue in the fragment (R400 for Hsp90α, Figure 1C) (Meyer et al., 2003, Prodromou, 2016), whereas K362-D372 is spatially adjacent to the catalytic loop (Figure 1C) and is found to indirectly modulate the ATP hydrolysis process by its intimate interactions with the catalytic loop in Hsp90's middle domain (Meyer et al., 2003). According to the [^1^H, ^15^N] HSQC titration data, the binding of **SOMCL-16-171** and **SOMCL-16-175** to Hsp90M only induce limited CSPs of the residues in the catalytic loop. However, significant CSPs for a few of residues in the region spanning K362-D372 were observed. Therefore, a detectable allosteric modulation effect on the ATPase activity of Hsp90α is expected. *In vitro* ATP hydrolysis assay with or without the presence of **SOMCL-16-175** was then conducted. The ATP hydrolysis process catalyzed by Hsp82 (Hsp90α homolog in yeast) was monitored by acquiring ^31^P spectra at different time points, and the representative spectra are shown in Figure 4E. At the reaction time point of 3 h after the initiation of the ATP hydrolysis, compared with the reaction system without the presence of **SOMCL-16-175**, higher concentrations of ADP and free phosphate ion and lower concentration of ATP were observed when **SOMCL-16-175** was added (Figures 4 and S9). And these data suggest that the ATPase activity of Hsp90α is promoted by the addition of **SOMCL-16-175**.

### SOMCL-16-171 and SOMCL-16-175 Interact with Hsp90 in Cellular Context and Cause Cytotoxicity in Human Breast Cancer Cell Lines

**Figure 5:**
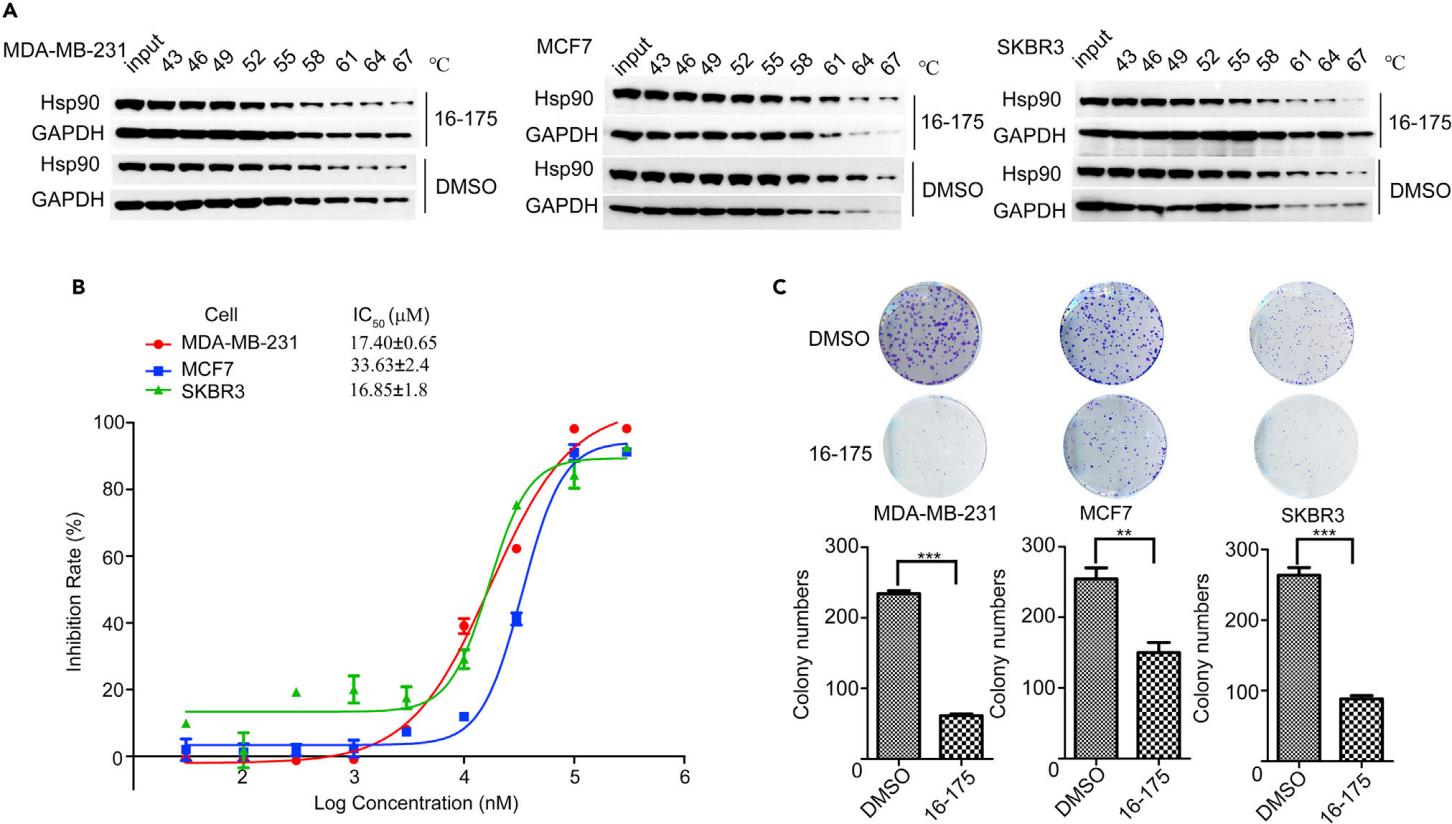
SOMCL-16-175 Interacts with Hsp90 in Cellular Context and Causes Cytotoxicity in Human Breast Cancer Cell Lines (A) Upon the treatment of **SOMCL-16-175**, decreased thermostability of Hsp90 in cellular context was observed. Extracts from MDA-MB-231, MCF7, and SKBR3 cells were used in the cellular thermal shift experiments. (B) Cell viability of MDA-MB-231, MCF7, and SKBR3 cells was assessed after exposure to vehicle and different concentrations of **SOMCL-16-175** (30, 100, and 300 nM and 1, 3, 10, 30, 100, and 300 μM) for 72 h. Data are analyzed by GraphPad Prism 5 and presented as means ± S.D. (n = 3). (C) Colony formation assays were performed on MDA-MB-231, MCF7, and SKBR3 cells treated with different concentrations of **SOMCL-16-175** (7.5 μM for MDA-MB-231 and SKBR3 cells, 17.5 μM for MCF7 cells) for 7–15 days, and quantitative results are shown in lower panel (n = 3, **p < 0.01 compared with control, ***p < 0.0001 compared with control, t test). See also Figure S12.

Hsp90 plays important roles in the development of cancers by modulating the maturation of cancer-related client proteins including transcription factors and kinase (Garg et al., 2016, Prodromou, 2009, Sidera and Patsavoudi, 2014). Besides, since Hsp90's chaperone cycle is intimately coupled to the sequential conformation changes induced by endogenous small molecules (ATP and ADP) and co-chaperones (p23, CDC37, Aha1), interfering with any one of the function-related structural states of Hsp90 would present modulation effects on its functional display, which might affect cell growth and proliferation. In this study, cellular thermal shift assay was used to confirm the interaction between **SOMCL-16-171/SOMCL-16-175** and Hsp90 in cellular context. In this assay, cell extracts from three breast cancer cell lines, including MDA-MB-231, MCF7, and SKBR3, were pre-incubated with either one of **SOMCL-16-171** and **SOMCL-16-175** or 1% DMSO for 20 min. The mixture samples were then submitted to a parallel incubation lasting for 5 min at different temperatures ranging from 43°C to 67°C. The level of Hsp90 in the after-incubation samples was detected and visualized by using immunoblotting technique (Figures 5A and S12). Compared with the treatment of DMSO, negative shifting of the stability of Hsp90 in the cellular context was observed upon pre-incubation with either **SOMCL-16-171** or **SOMCL-16-175** (Figures 5A, S10, and S12), which suggests that both **SOMCL-16-171** and **SOMCL-16-175** could interact with Hsp90 in the cellular context. After the cellular thermal shift assay, cell viability assay and colony formation experiment were applied to test if the application of **SOMCL-16-171** and **SOMCL-16-175** would cause cytotoxicity. Three breast cancer cell lines, including MDA-MB-231, MCF7, and SKBR3, were used in the experiments. Both the cell viability data and the colony formation results indicate that **SOMCL-16-171** and **SOMCL-16-175** could inhibit cell growth and proliferation (Figures 5 and S10). Moreover, in comparison with **SOMCL-16-171**, the compound **SOMCL-16-175** presents stronger inhibition effects on all of the three cell lines, and the IC50s of **SOMCL-16-175** are 16.48, 34.67, and 13.96 μM for MDA-MB-231, MCF7, and SKBR3, respectively (Figure 5). It is worth noting that the cellular inhibition activity data for **SOMCL-16-171** and **SOMCL-16-175** are consistent with their capacities for down-regulating the thermal stability of Hsp90. With the addition of **SOMCL-16-171** and **SOMCL-16-175**, the *Tm* shifts of Hsp90NMΔ were determined to be −0.79°C and −6.27°C, respectively (Figure 3).

### SOMCL-16-175 Promotes the ATPase Activity of Hsp90 and Destabilizes Its Client Proteins

**Figure 6:**
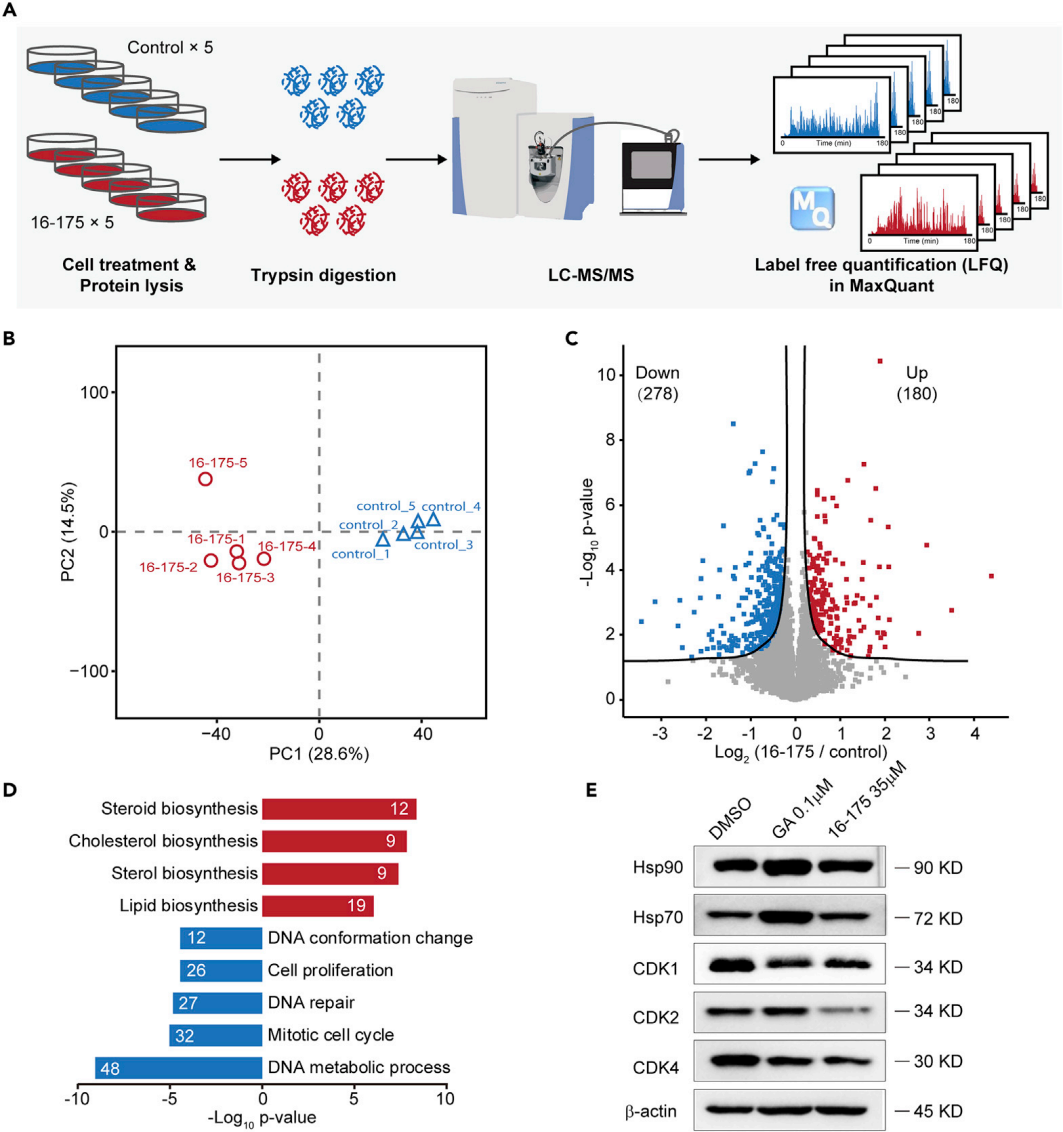
Down-Regulation of the Cell-Cycle Process Revealed by Quantitative Proteomic Analysis (A) Workflow of the quantitative proteomic analysis. MCF7 cells treated by DMSO control or **SOMCL-16-175** for 48 h were lysed for protein extraction (five replicates for each condition). Peptides were prepared using the FASP (filter-aided sample preparation) method and then subjected to LC-MS/MS analysis using a Q Exactive HF mass spectrometer. Label-free quantification (LFQ) in MaxQuant software was used for relative proteomic quantification. (B) Principle component analysis (PCA) of the proteomic data for sample replicates. Control and **SOMCL-16-175** samples are clearly separated in PC1, suggesting the differences in proteome between control and **SOMCL-16-175** samples. (C) Volcano plot reveals the significantly up-regulated and down-regulated proteins in **SOMCL-16-175**-treated samples compared with control, using a global permutation-based FDR approach implemented in Perseus software. (D) Highly over-represented biological processes in the up-regulated and down-regulated proteins by Fisher's exact test. Protein counts belonging to each process were labeled on the bars. (E) Consistent with the proteomic analysis results, the immunoblot data also indicate that **SOMCL-16-175** destabilizes Hsp90's client proteins but does not trigger significant heat-shock response. See also Figure S12.

To unravel the potential molecular mechanisms underlying the modulation effects of **SOMCL-16-175** on Hsp90, we analyzed the proteomic changes of MCF7 cells upon the presence of **SOMCL-16-175** by the label-free quantification (LFQ)-based quantitative proteomic method (Figure 6A). Totally, we identified 51,393 peptide sequences, corresponding to 4,866 proteins, with an average of ~30,000 peptide sequences and ~4,500 proteins in each sample (Figures S11A and S11B). The LFQ intensities of identified proteins were distributed consistently and correlation coefficients among different samples are 0.97 on average, both demonstrating the high quality of our MS data (Figures S11C and S11D). Principle component analysis (PCA) of the proteomic data indicates a clear separation between the control group and the **SOMCL-16-175**-treated group (Figure 6B). By a global permutation-based FDR approach (Tusher et al., 2001), a total of 458 proteins (up, 180; down, 278) have been revealed to be regulated upon the addition of **SOMCL-16-175** (Figure 6C). Notably, DNA function regulation and cell-cycle-related biological processes are over-represented in the down-regulated proteins (Figure 6D), suggesting the cell cytotoxicity of **SOMCL-16-175**. Quite a few of down-regulated proteins are known clients of Hsp90 and are summarized in Table 2. Among the affected client proteins of Hsp90 (Table 2), the levels of CDK1 (cyclin-dependent kinase 1) and CDK2 (cyclin-dependent kinase 2), which are two of the key players in cell-cycle control, were further tested by using immunoblot approach. Consistent with the proteomic data, significant down-regulation of CDK1 and CDK2 in response to **SOMCL-16-175** treatment was observed (Figures 6E and S12). Meanwhile, CDK4 (cyclin-dependent kinase 4), one of kinase clients of Hsp90, also presents modest decrease in its cellular levels upon the addition of the compound (Figures 6E and S12). Interestingly, although the application of **SOMCL-16-175** destabilizes Hsp90's client proteins (Table 2, Figures 6E and S12), no significant heat-shock response is triggered. Only a minor up-regulation was observed for the level of Hsp70 and Hsp90 upon the treatment of the compound (Figures 6E and S12). Overall, the observed down-regulation of Hsp90's client proteins suggests that the working cycle of the chaperone is finely tuned and the allosteric modulation of the function display of Hsp90 could be achieved by targeting its middle domain.

**Table 2:** Summary of the Down-Regulated Client Proteins of Hsp90 in MCF7 Cells with the Presence of SOMCL-16-175

Uniprot	Protein	References
Q6PJG6	BRAT1 (BRCA1 associated ATM activator 1)	Fierro-Monti et al., 2013
P06493	CDK1 (cyclin-dependent kinase 1)	Garcia-Morales et al., 2007
P24941	CDK2 (cyclin-dependent kinase 2)	Prince et al., 2005
P26358	DNMT1 (DNA (cytosine-5)-methyltransferase 1)	Zhou et al., 2008
Q9Y6Y0	IVNS1ABP (influenza virus NS1A-binding protein)	Zhang et al., 2011
Q99538	LGMN (protein encoded by the LGMN gene)	Lin et al., 2014
Q02750	MAP2K1 (dual specificity mitogen-activated protein kinase kinase 1)	Stancato et al., 1997
P15941	MUC1 (mucin 1)	Ren et al., 2006
Q6P4R8	NFRKB (nuclear factor related to kappa-B-binding protein)	Taipale et al., 2012
P12004	PCNA (proliferating cell nuclear antigen)	Wang et al., 2010
Q96T88	UHRF1 (ubiquitin like with PHD and ring finger domains 1)	Ding et al., 2016

## Discussion

Hsp90 belongs to the chaperone superfamily and plays crucial roles in maintaining the stability and the activity of numerous client proteins, including kinases, transcription factors, and steroid hormone receptors*.* Owing to its function in controlling the cellular homeostasis of cancer-related proteins such as B-Raf (protein kinase encoded by the BRAF gene), CDK4, and v-src (tyrosine kinase encoded by the v-Src gene), Hsp90 has emerged as a promising target for anti-cancer drug development (Garg et al., 2016, Prodromou, 2009, Sidera and Patsavoudi, 2014). However, although quite a few of candidate compounds targeting the canonical ATP binding pocket in Hsp90's N-terminal domain have stepped into the clinical trial stage, none of them has been approved for cancer therapy. There are multiple reasons that hinder the potential use of Hsp90N-targeted ATP competitive inhibitors in practice. Limited efficacy, pro-survival heat shock response, and poor binding selectivity over Hsp90's different isoforms compromise their therapeutic potential (Garg et al., 2016, Vartholomaiou et al., 2016). Therefore, developing non-ATP competitive compounds targeting Hsp90 deserves to be tested. In fact, quite a few of research works published during past several years indicate that the allosteric modulators binding to either the N-terminal domain or the interface region in between the middle domain and the C-terminal domain of Hsp90 might serve as new potential therapeutic opportunities (Bassanini et al., 2018, D'Annessa et al., 2017, Ferraro et al., 2019, Sattin et al., 2015, Yokoyama et al., 2015, Zierer et al., 2016, Zierer et al., 2014). Different from the working mode of the canonical ATP competitive inhibitors of Hsp90, the allosteric modulators target the non-canonical binding sites of the chaperone and induce activation of its ATPase activity (D'Annessa et al., 2017, Roe et al., 2018, Sattin et al., 2015, Yokoyama et al., 2015, Zierer et al., 2014). Meanwhile, although presenting accelerating effect on Hsp90's ATP hydrolysis activity, the application of these allosteric modulators would down-regulate the chaperoning function of Hsp90 for client proteins and cause cytotoxicity (D'Annessa et al., 2017, Ferraro et al., 2019, Sattin et al., 2015, Yokoyama et al., 2015, Zierer et al., 2014). These results indicate that the ATPase activity does not fully correlate with the chaperoning activity of Hsp90 (Ferraro et al., 2019). The delicately tuned conformational events and their associated time schedule are both required for the fully functional display of the chaperone. Therefore, both the ATPase inhibitors and the ATPase accelerators would down-regulate Hsp90's cellular activity through perturbing the timing of its chaperoning cycle.

Here in this manuscript, non-covalent allosteric modulators **SOMCL-16-171** and **SOMCL-16-175** targeting Hsp90α’s middle domain were discovered by a combination use of experimental screening and medicinal chemistry-guided optimization. Compared with Hsp90α’s N-terminal domain, which has a conserved ATP binding pocket shared by GHL-ATPase subfamily members (Dutta and Inouye, 2000), Hsp90α’s middle domain mainly functions in client protein recognition (Meyer et al., 2003, Prodromou and Pearl, 2003) and presents no known binding cavity for small molecules. Up to date, only active compounds covalently bonding to the cysteine residue in the middle domain of Hsp90α have been reported. Therefore, fragment-based lead discovery approach, which has been proved to serve as a powerful tool for active compound discovery targeting allosteric binding sites and protein-protein interactions, was applied for Hsp90α’s middle domain-targeted hit compound screening. Fortunately, one hit compound **1-E6** from a fragment pool containing 539 compounds was screened out (Figures 2 and S2). After that, medicinal chemistry-guided hit compound optimization was carried out and tens of compounds were synthesized. However, among these compounds, only **SOMCL-16-171** and **SOMCL-16-175** present enhanced CSP effects when binding to Hsp90M (Figure 2). Besides, we found that the optimization attempt through fragment expansion would decrease the binding capacity of the compound to Hsp90M. It suggests that the volume of the binding pocket in Hsp90M for **1-E6** is quite limited. And this finding is consistent with the aforementioned physiological function of Hsp90α’s middle domain. In the following studies, multiple techniques including NMR, ITC, and thermal shift assay were used to confirm and characterize the binding of **SOMCL-16-171** and **SOMCL-16-175** to Hsp90α’s middle domain. These two compounds were demonstrated to allosterically modulate the conformation of Hsp90α’s N-terminal domain, which consequentially affects the thermodynamics of Hsp90N's interactions with ATP (AMPPNP) and ADP (Figure 3). The CSP analysis data, the mutagenesis study results, and the generated docking model indicate that **SOMCL-16-171** and **SOMCL-16-175** bind to the pocket composed of one α-helix and two loops including K443-E451, F349-N360, and D372-G387 (Figures 4 and S8). And as revealed by the NMR data, loop region F349-K356 undergoes slow conformational exchange in solution, and the resonances for amino acid residues from F349 to K356 are totally absent in the recorded NMR spectra. The high flexibility of F349-K356 is expected to favor the recognition of the active compounds by Hsp90α’s middle domain. Meanwhile, as revealed by the CSP analysis data, the binding of **SOMCL-16-175** would induce long-range allosteric modulation effect on the hydrophobic region spanning K362-D372 of Hsp90α’s middle domain (Figure 4). Since K362-D372 fragment has been reported to play a role in driving the function-related conformational changes of the catalytic loop in Hsp90's middle domain (Figure 1) (Meyer et al., 2003), we then expect that the binding of **SOMCL-16-171** and **SOMCL-16-175** might modulate the ATPase activity of Hsp90α. This expectation is confirmed by the result of the *in vitro* ATP hydrolysis assay. With the addition of **SOMCL-16-175**, Hsp82 (yeast homolog of Hsp90α) presents an enhancement in its ATPase activity (Figure 4).

As mentioned earlier, the developed allosteric modulators **SOMCL-16-171** and **SOMCL-16-175** could accelerate the ATPase activity of Hsp90 by binding to the pocket composed of F349-N360, K443-E451, and D372-G387 in the middle domain of the chaperone (Figure 4). As it has been known, the co-chaperone Aha1 could stimulate the ATPase activity of Hsp90. The working mode for Aha1:Hsp90 system includes the binding of Aha1's N-terminal domain to Hsp90's middle domain and the nucleotide-dependent binding of Aha1's C-terminal domain to Hsp90's N-terminal domain, which occur in a sequential manner (Retzlaff et al., 2010). A very recent study indicates that the phosphorylation on Y313 of Hsp90's middle domain, which has been reported to promote the recruitment of Aha1 (Xu et al., 2012), would enhance the formation of a transient complex in which both the N-terminal and C-terminal domains of Aha1 bind independently to distinct surfaces of the middle domains in opposing Hsp90 protomers (Xu et al., 2019). Besides, the perturbed residues in the middle domain of Hsp90 Y313E (a phosphomimetic mutation) mutant upon the binding of Aha1 are identified to be I361, I370, I378, I385, I445, I491, I494, and I519 (Xu et al., 2019). Since a few of these residues fall into the **SOMCL-16-171** and **SOMCL-16-175** recognition regions including K443-E451 and D372-G387 in Hsp90's middle domain, binding of the compounds to the chaperone might potentially interfere with the recruitment of Aha1. However, owing to the significant difference in the binding affinities for Aha1 and the developed compound **SOMCL-16-175** to Hsp90, which are 13 (Xu et al., 2012) and 804 μM, respectively, the possible interfering effects are not observable in this study. An allosteric modulator with significantly enhanced binding affinity to the identified pocket in Hsp90's middle domain could serve as a probe molecule to answer the aforementioned question and provide more information for a further understanding of the chaperoning cycle of the chaperone.

After the *in vitro* characterization of the interactions between Hsp90α and the active compounds targeting Hsp90α’s middle domain, *in vivo* assays including cell viability assay, colony formation experiment, and mass spectroscopy-based proteomics analysis were carried out to test the cellular activities of the compounds and reveal the underlying molecular mechanisms coupled to their *in vivo* activities. Not surprisingly, both **SOMCL-16-171** and **SOMCL-16-175** cause cytotoxicity in human breast cancer cell lines MDA-MB-231, MCF7, and SKBR3 (Figures 5 and S10). Besides, we found that, among two active compounds, **SOMCL-16-175** shows stronger cytotoxicity than **SOMCL-16-171** (Figures 5 and S10). This is consistent with their *in vitro* characterization data. The binding of **SOMCL-16-175** would induce a much larger *Tm* shift of Hsp90α (Figures 3 and S7). Since **SOMCL-16-175** presents stronger cytotoxicity in breast cancer cell lines, a further proteomics study was applied to investigate the potential working mechanisms linking to the anti-proliferation activity of this compound. Upon the treatment of **SOMCL-16-175**, the down-regulation of the cell-cycle process in MCF7 cell line was revealed by the quantitative proteomic analysis and the decreased levels of multiple key players involved in the cell-cycle pathway were observed (Figure 6). More interestingly, both the proteomic analysis and the immunoblot data demonstrate that no significant heat-shock response is triggered by the treatment of **SOMCL-16-175** (Figures 6 and S12). Although the modest binding of the compound to Hsp90 might potentially contribute to this observation, it is still an encouraging finding and deserves to be investigated further. To achieve an exclusive conclusion, allosteric modulator with stronger binding affinity to Hsp90α’s middle domain needs to be developed and evaluated.

### Limitations of the Study

In this study, two allosteric modulators (**SOMCL-16-171** and **SOMCL-16-175**) of Hsp90α were developed. However, these two compounds only present modest binding capabilities to the middle domain of the chaperone. And at least partially due to the low binding affinities, no high-resolution complex structures for Hsp90M:**SOMCL-16-171** and Hsp90M:**SOMCL-16-175** were solved, which would make a further compound optimization derived from **SOMCL-16-171**/**SOMCL-16-175** less efficient.

## Methods

All methods can be found in the accompanying Transparent Methods supplemental file.

## Data and Code Availability

The accession number for the atomic coordinates of Hsp90M protein reported in this paper is PDB: 6KSQ.

## Supplemental Information

Document S1. Transparent Methods, Figures S1–S12, and Table S1
