# Supplementary material for: Allosteric Regulation of Hsp90α’s Activity by Small Molecules Targeting the Middle Domain of the Chaperone
Source: iScience. 2020 Jan 21;23(2):100857. doi: 10.1016/j.isci.2020.100857 (PMC6997908; doi:10.1016/j.isci.2020.100857)
Supplement: Document S1. Transparent Methods, Figures S1–S12, and Table S1 [file mmc1.pdf]

iScience, Volume *23*

Supplemental Information

Allosteric Regulation of Hsp90α's Activity by Small Molecules Targeting the Middle Domain of the Chaperone

Chen Zhou, Chi Zhang, Hongwen Zhu, Zhijun Liu, Haixia Su, Xianglei Zhang, Tingting Chen, Yan Zhong, Huifang Hu, Muya Xiong, Hu Zhou, Yechun Xu, Ao Zhang, and Naixia Zhang

Supplemental Figures

Figure S1

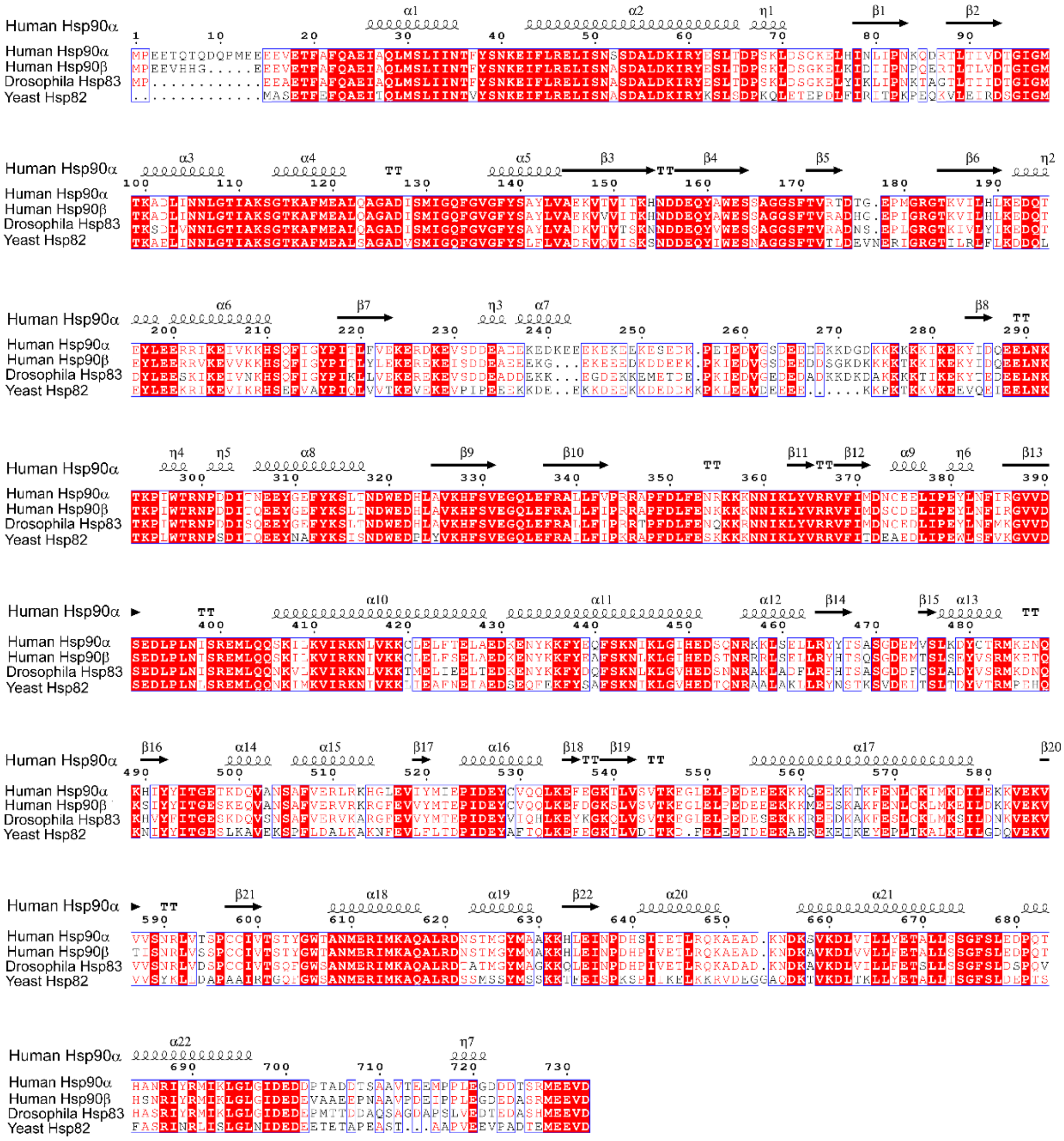

Figure S1. Sequence alignment of representative cytosolic Hsp90 homologs from different species. Related to Figure 1. Conserved residues are marked in red.

Figure S2

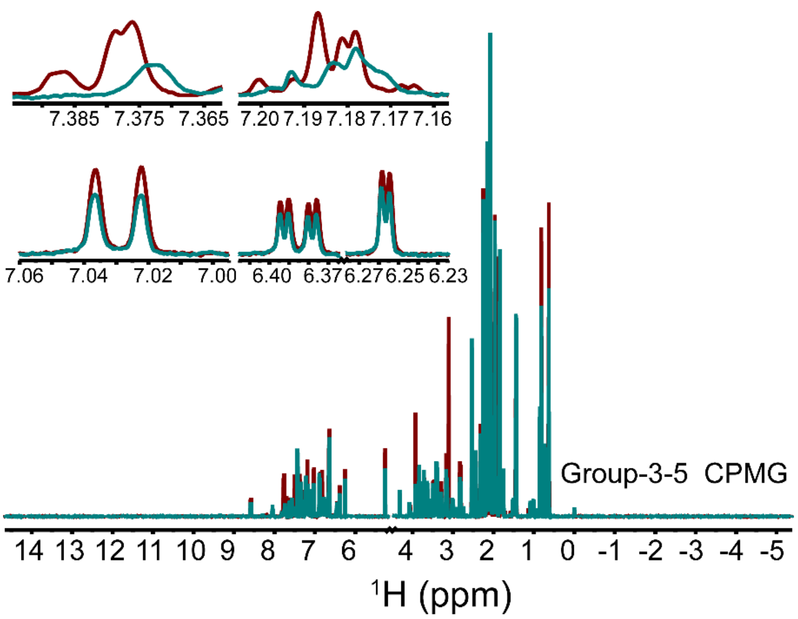

Figure S2. Ligand observed CPMG spectra indicate that 1-E6 in Group-3-5 compound mixture potentially interacts with Hsp90α's middle domain. Related to Figure 2. CPMG NMR spectra for Group-3-5 compound mixture only (200 μM) and Group-3-5 compound mixture (200 μM) with the presence of Hsp90α middle domain (5 μM) are colored in red and cyan, respectively.

Figure S3

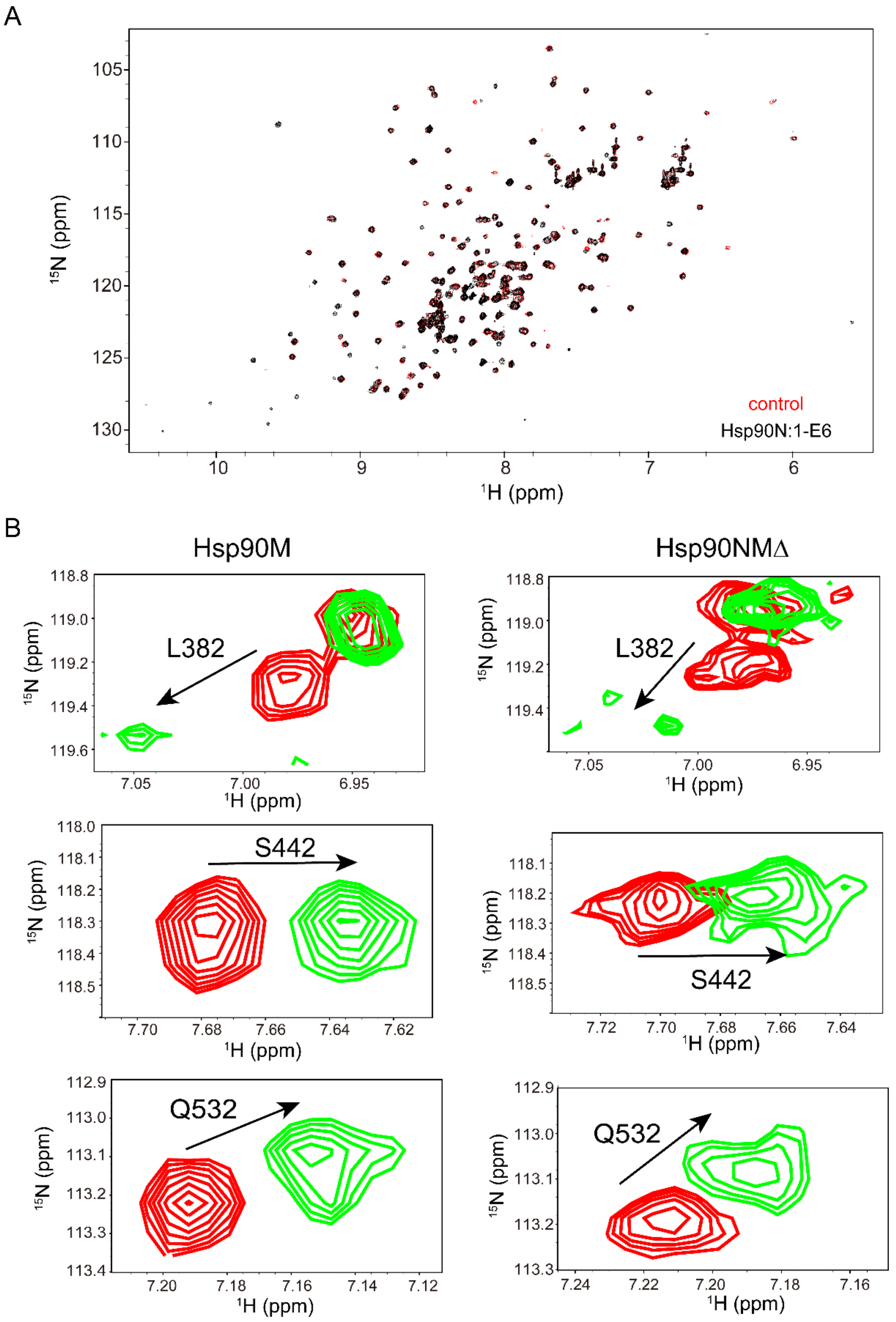

Figure S3. Hit compound 1-E6 has a weak interaction with Hsp90α's N-terminal domain. Related to Figure 2. (A) Superposition of [^{1}H, ^{15}N] HSQC spectra of Hsp90α's N-terminal domain without (red) and with **1-E6** (black, molar ratio of 1:4 Hsp90α N-terminal domain to **1-**

E6) reveals a minor spectral change upon the addition of the compound. (B) The specific interactions between **1-E6** and Hsp90α's middle domain were confirmed by [^{1}H, ^{15}N] HSQC titration experiments. [^{1}H, ^{15}N] HSQC experiments recorded on Hsp90α's middle domain and Hsp90NMΔ with its middle domain ^{15}N labelled without (red) or with the presence of **1-E6** (green) reveal significant spectral changes when the compound is present.

Figure S4

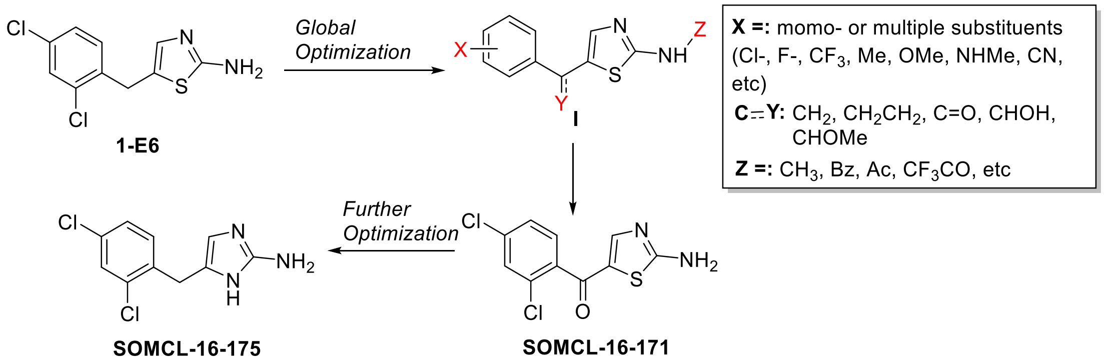

Figure S4. Medicinal chemistry optimization of the hit compound 1-E6. Related to Figure 2.

Figure S5

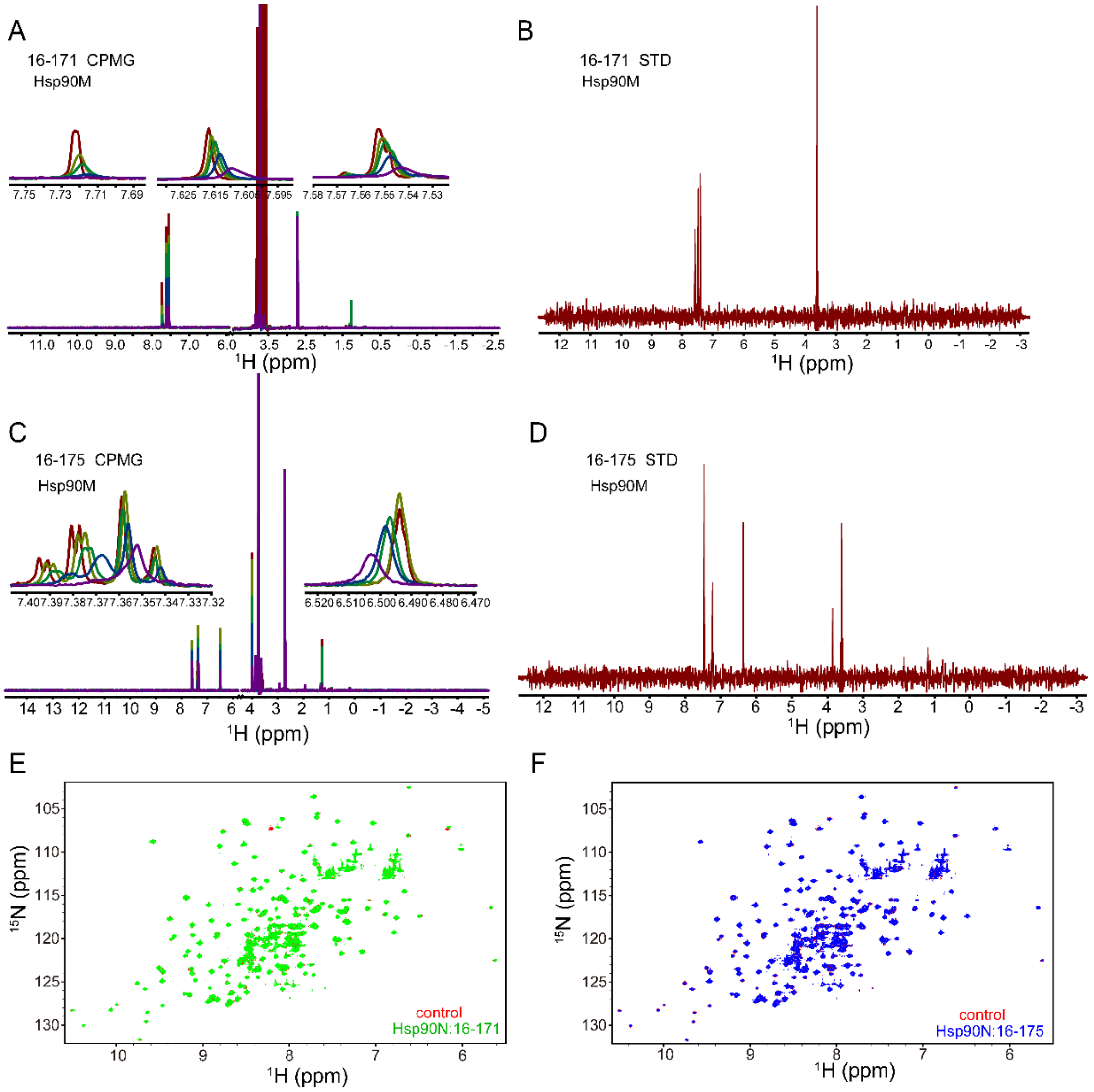

Figure S5. Ligand observed CPMG and STD spectra indicate that SOMCL-16-171 and SOMCL-16-175 directly interact with Hsp90α's middle domain. Related to Figure 2. (A) CPMG NMR spectra for **SOMCL-16-171** only (200 μM, red) and **SOMCL-16-171** (200 μM) in the presence of different concentrations of Hsp90α middle domain (2 μM, lime-green; 5 μM, green; 10 μM, blue; 20 μM, purple). (B) STD spectrum of **SOMCL-16-171** (200 μM) in the presence of Hsp90α middle domain (5 μM). (C) CPMG NMR spectra for **SOMCL-16-175** only (200 μM, red) and **SOMCL-16-175** (200 μM) in the presence of different concentrations of Hsp90α middle domain (2 μM, lime-green; 5 μM, green; 10 μM, blue; 20 μM, purple). (D) STD spectrum of **SOMCL-16-175** (200 μM) in the presence of Hsp90α middle domain (5 μM). (E) Superposition of [^{1}H, ^{15}N] HSQC spectra of Hsp90α's N-terminal domain without (red) and

with **SOMCL-16-171** (green, molar ratio of 1:4 Hsp90α N-terminal domain to **SOMCL-16-171**) reveals a non-significant spectral change upon the addition of the compound. (F) Superposition of [^{1}H, ^{15}N] HSQC spectra of Hsp90α's N-terminal domain without (red) and with **SOMCL-16-175** (blue, molar ratio of 1:4 Hsp90α N-terminal domain to **SOMCL-16-175**). No spectral change upon the addition of **SOMCL-16-175** was detected.

Figure S6

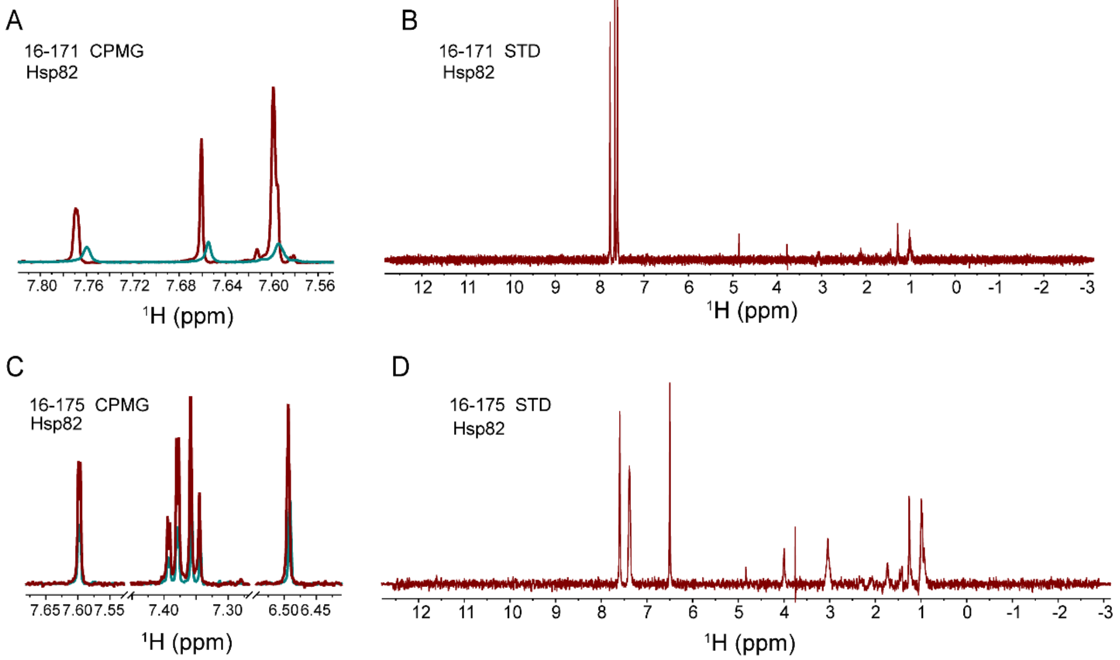

Figure S6. Ligand observed CPMG and STD spectra indicate that SOMCL-16-171 and SOMCL-16-175 directly interact with Hsp82 (Hsp90α yeast homolog). Related to Figure 2. (A) CPMG NMR spectra for **SOMCL-16-171** (200 μM, red) and **SOMCL-16-171** (200 μM) in the presence of Hsp82 (5 μM, green). (B) STD spectrum of **SOMCL-16-171** (200 μM) in the presence of Hsp82 (5 μM). (C) CPMG NMR spectra for **SOMCL-16-175** (200 μM, red) and **SOMCL-16-175** (200 μM) in the presence of Hsp82 (5 μM, green). (D) STD spectrum of **SOMCL-16-175** (200 μM) in the presence of Hsp82 (5 μM).

Figure S7

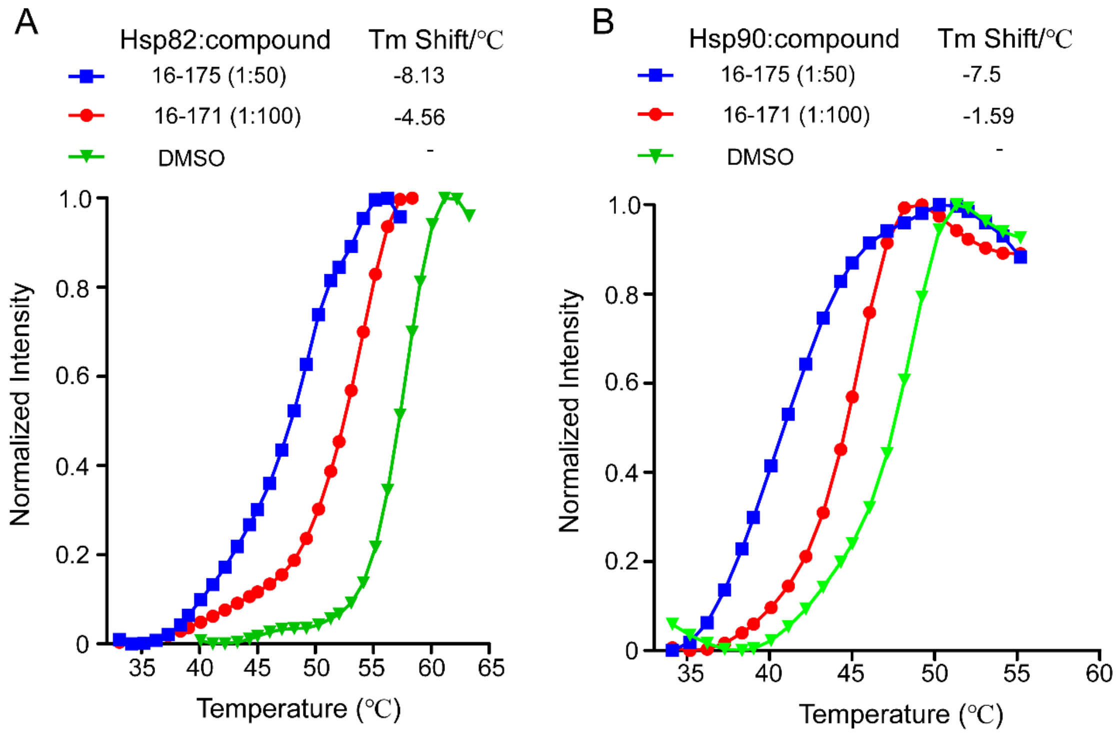

Figure S7. SOMCL-16-175 and SOMCL-16-171 directly interact with Hsp90α and its yeast homolog Hsp82. Related to Figure 3. (A, B) The shifts in *Tm* values of full-length Hsp90 (human Hsp90α), full-length Hsp82 (Hsp90α yeast homolog) upon the binding of **SOMCL-16-171** or **SOMCL-16-175** were determined.

Figure S8

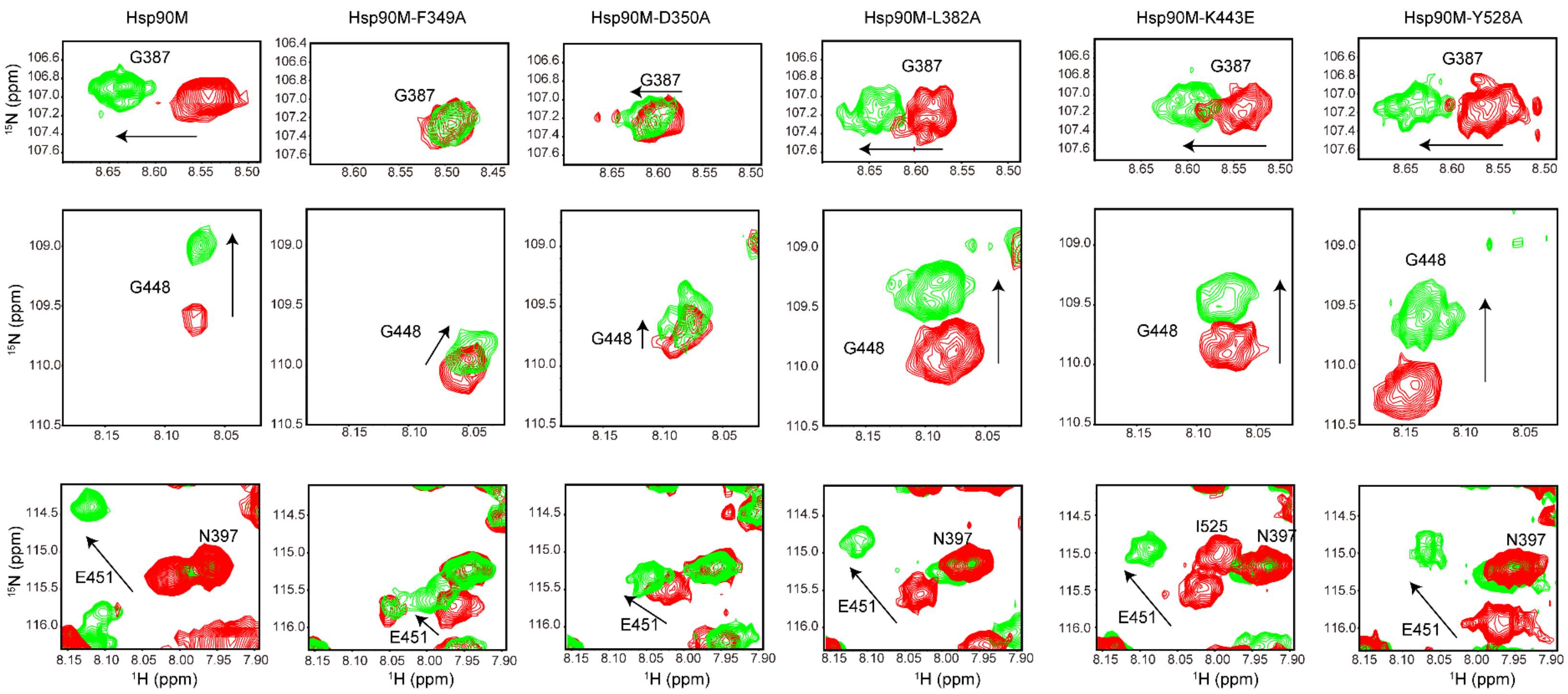

Figure S8. Compared with the wild-type chaperone, Hsp90M mutants with the mutation of representative residues potentially responsible for the recognition of active compounds present less significant or comparable chemical shift perturbations upon the addition of SOMCL-16-175. Related to Figure 4. Superposition of [^{1}H, ^{15}N] HSQC spectra of Hsp90M/Hsp90M-F349A/Hsp90M-D350A /Hsp90M-L382A/Hsp90M-K443E/Hsp90M-Y528A without (red) and with the presence of **SOMCL-16-175** (green, molar ratio of 1:6 Hsp90M or its mutants to **SOMCL-16-175**) reveals chemical shift perturbation effects upon active compound binding.

Figure S9

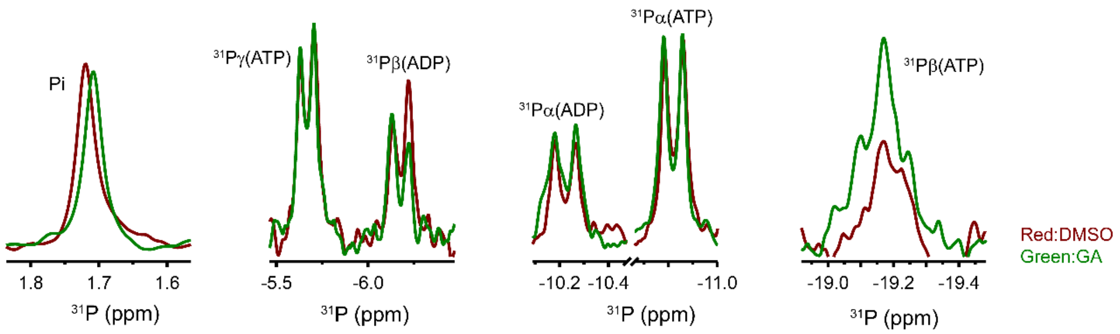

Figure S9. The ^{31}P NMR spectra data suggest that the application of Geldanamycin (known Hsp90 inhibitor binding to Hsp90's N-terminal domain) inhibits the ATPase activity of Hsp82. Related to Figure 4. The ATP hydrolysis process catalyzed by Hsp82 (Hsp90α yeast homolog) was monitored by acquiring 1D ^{31}P spectra at different reaction time points. Superposition of 1D ^{31}P spectra of Hsp82:ATP (3 μM:1mM) reaction system without (red) and with the presence of Geldanamycin (200 μM, green) acquired at the time point of 3 hours after the initiation of the reaction.

Figure S10

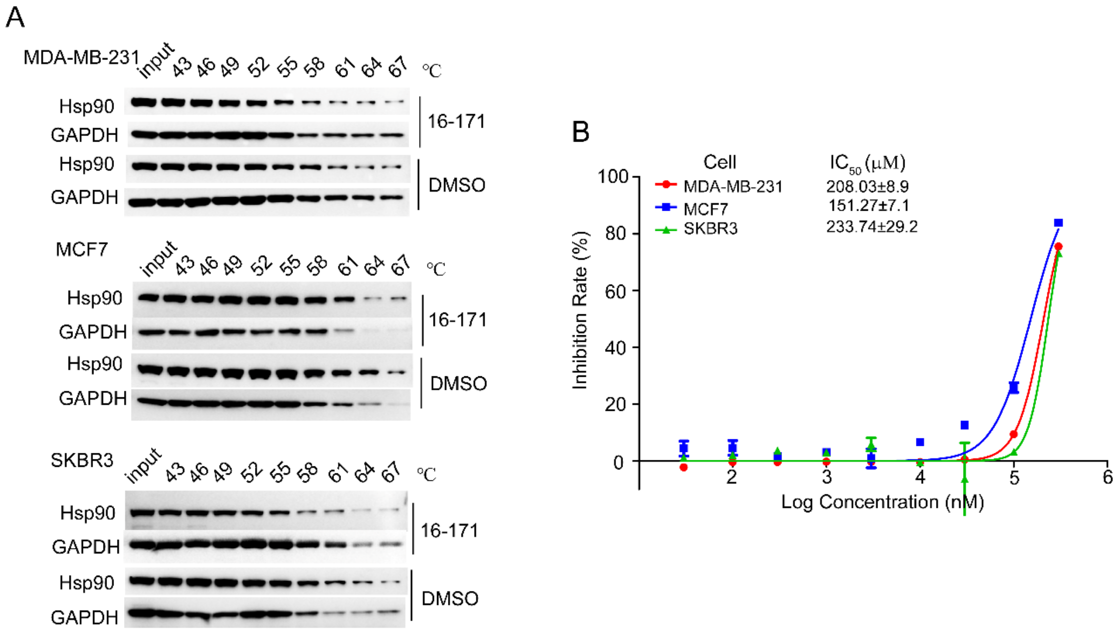

Figure S10. SOMCL-16-171 interacts with Hsp90 in cellular context and cause cytotoxicity in human breast cancer cell lines. Related to Figure 5. (A) Upon the treatment of **SOMCL-16-171**, decreased thermostability of Hsp90 in cellular context was observed. Extracts from MDA-MB-231, MCF7 and SKBR3 cells were used in the cellular thermal shift experiments. (B) Cell viability of MDA-MB-231, MCF7 and SKBR3 cells were assessed after exposure to vehicle, and different concentrations of **SOMCL-16-171** (30 nM, 100 nM, 300 nM, 1 μM, 3 μM, 10 μM, 30 μM, 100 μM, 300 μM) for 72 hours. Data are analyzed by GraphPad Prism 5 and presented as means ± S.D. (n = 3).

Figure S11

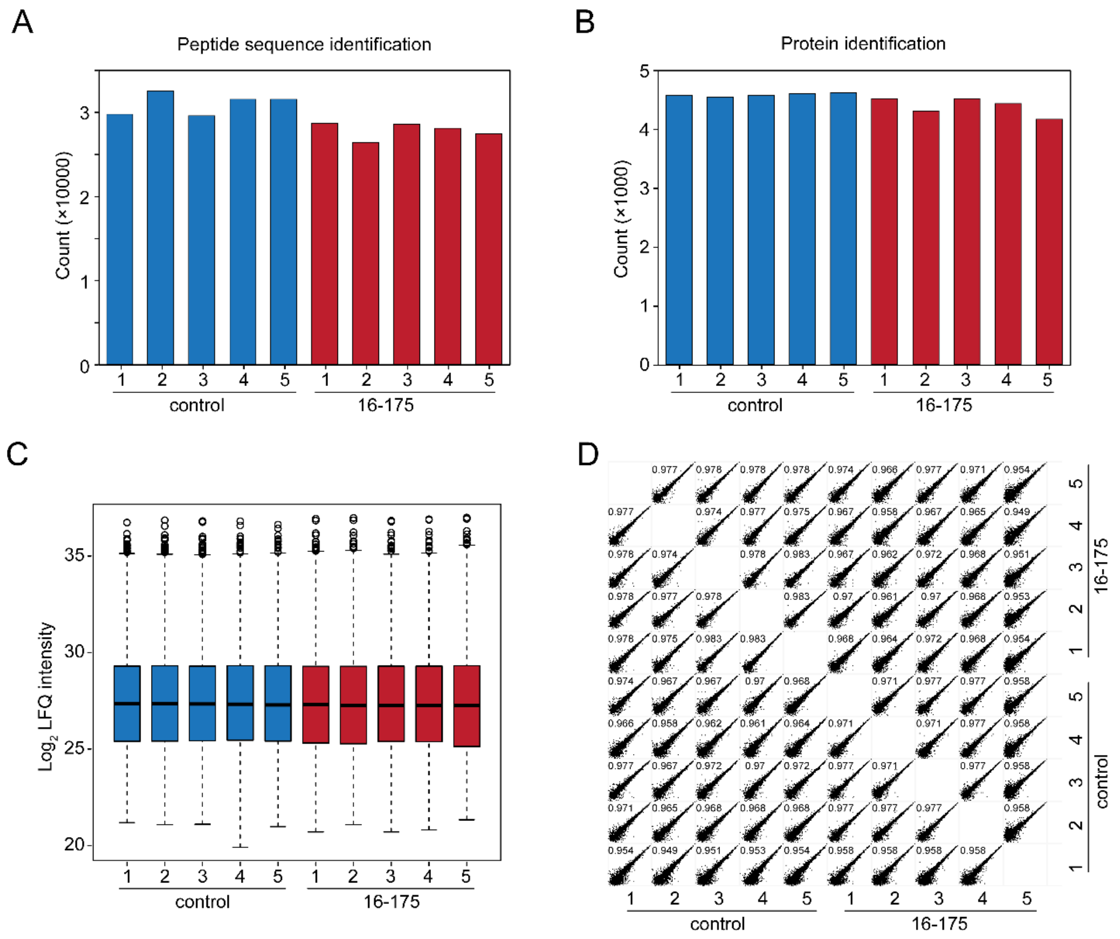

Figure S11. Summary of the proteomic data. Related to Figure 6. (A) Counts of peptide sequence identification. Totally, 51,393 peptide sequences were identified, with averagely ~30,000 in each sample. (B) Counts of protein identification. Totally, 4,866 proteins were identified, with averagely ~4,500 proteins in each sample. (C) Boxplot shows the distributions of log2-transformed LFQ intensities in each sample. (D) The quantification correlation plot. Pearson coefficients were calculated using all quantified proteins for each pair of samples.

Figure S12

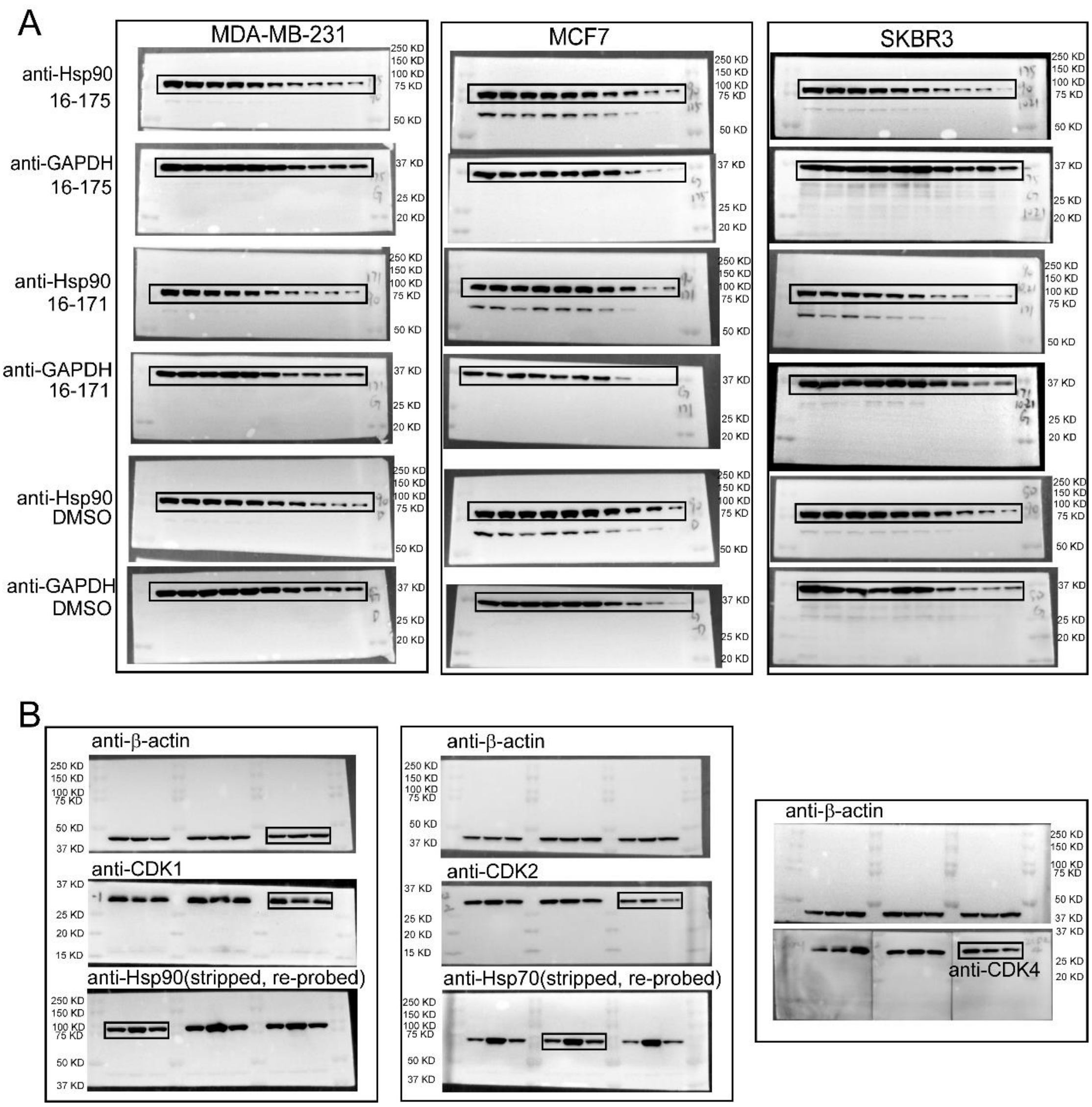

Figure S12. Uncropped western blot images shown in Figure 5A, supplemental Figure S10A and Figure 6E. (A) Related to Figure 5 and supplemental Figure S10. The cropped regions are framed. (B) Related to Figure 6. The cropped regions are framed.

Supplemental Table

Table S1

Table S1. Crystallography data collection and refinement statistics. Related to Figure 1.

**PDB ID**	6KSQ	**RMS values**	
Space group	*P*21	Bond length (Å)	0.008
Cell dimension: a (Å)	36.07	Bond angle (°)	0.867
b (Å)	38.97	**Numbers of non-hydrogen atoms**	
c (Å)	108.03	Protein	2094
Wavelength (Å)	0.979	Water Oxygen	62
Reflections (unique)	14524	Others	0
Resolution range (Å)	2.20-36.64	**Mean temperature factors (Å^2)**	
Highest-resolution shell (Å)	2.20-2.24	Protein	45.35
Redundancy	6.30	**Ramachandran plot**	
I/σ (I)	8.50	Favored (%)	98.41
Completeness (%)	94.50	Allowed (%)	1.59
Rwork/Rfree	0.182/0.232	Outliers (%)	0.00

Synthesis of compound SOMCL-16-171 and SOMCL-16-175

Synthesis of compound SOMCL-16-171

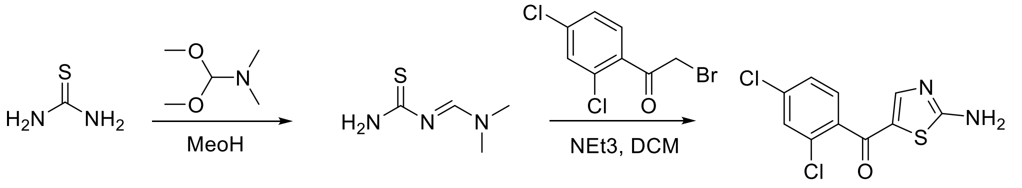

1-1 SOMCL-16-171

(E)-*N′*-Carbamothioyl-*N,N*-dimethylformimidamide (1-1):

To a solution of thiourea (760 mg, 10 mmol) in menthol was added N,N-dimethylformamide dimethyl acetal (1.3 equiv). The mixture was heated at 80 °C for 4 h. Then the mixture was concentrated and purified with column chromatography to afford the title compound as light yellow solid in 46%. **^{1}H NMR** (300 MHz, $CDCl_3$) δ 8.78 (s, 1H), 3.75 (m, 2H), 3.21 (s, 3H), 3.10 (s, 3H).

(2-Aminothiazol-5-yl)(2,4-dichlorophenyl)methanone (SOMCL-16-171):

To a solution of **1-1** (200 mg, 1.527 mmol) in CH_2Cl_2 were added 2-bromo-1-(2,4-dichlorophenyl)ethanone (1 equiv) and triethylamine (2 equiv). The mixture was stirred at room temperature overnight. Then the mixture was concentrated, and the residue obtained was purified with column chromatography to yield **SOMCL-16-171** as yellow solid in 40%. **^{1}H NMR** (300 MHz, $CDCl_3$) δ 7.50 (s, 1H), 7.40 – 7.31 (m, 3H), 6.06 (s, 2H); **^{13}C NMR** (151 MHz, $CDCl_3$) δ 184.39, 174.80, 151.23, 136.67, 136.28, 132.29, 130.26, 129.78, 129.75, 127.01; **ESI-MS**: 271 $[M - H]^+$; **HRMS-ESI**: *m/z* $[M - H]^+$ calcd for $C_{10}H_5Cl_2N_2OS$: 270.9500, found: 270.9508.

Synthesis of compound SOMCL-16-175

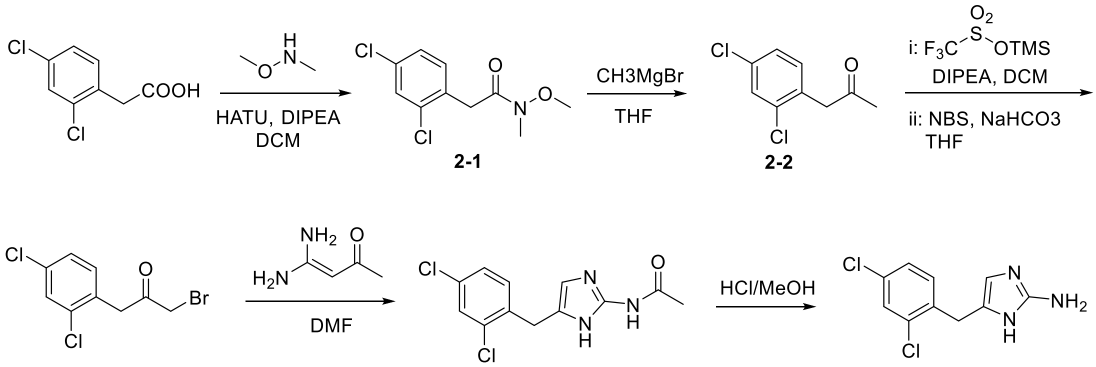

2-1 2-2 2-3 2-4 SOMCL-16-175

2-(2,4-Dichlorophenyl)-*N*-methoxy-N-methylacetamide (2-1):

To a solution of 2-(2,4-dichlorophenyl)acetic acid (205 mg, 1 mmol) in CH_2Cl_2 were added N,O-dimethylhydroxylamine (1.3 equiv), 1-(3-dimethylaminopropyl)-3-ethylcarbodiimide hydrochloride (EDCI, 1.3 equiv) and 1-hydroxybenzotriazole (HOBT, 1.3 equiv). Di(isopropyl)ethylamine (DIPEA, 3 mL) was added. The mixture was stirred at room temperature for 2 h, and then concentrated. The residue was purified with column chromatography to yield the title compound as colorless oil in 80% yield. **^{1}H NMR** (300 MHz, $CDCl_3$) δ 7.39 (s, 1H), 7.25–7.18 (m, 2H), 3.87 (s, 2H), 3.71 (s, 3H), 3.21 (s, 3H).

1-(2,4-Dichlorophenyl)propan-2-one (2-2):

A solution of **2-1** (197 mg, 0.798 mmol) in THF cooled to -78 ºC was added to the solution of N-acetylguanidine (0.86 mL, 1.4 M) in MeOH/toluene. The mixture was stirred at -78 °C for 30 min, followed by stirring at room temperature for 1 h. The reaction was quenched with saturated aqueous NH_4Cl solution, extracted with ethyl acetate, concentrated and purified with column chromatography to yield the title compound as colorless oil in 77% yield. **^{1}H NMR** (300 MHz, $CDCl_3$) δ 7.41 (s, 1H), 7.22 (d, J = 8.2 Hz, 1H), 7.14 (d, J = 8.2 Hz, 1H), 3.82 (s, 2H), 2.22 (s, 3H).

1-Bromo-3-(2,4-dichlorophenyl)propan-2-one (2-3):

DIPEA (1.2 equiv) and trimethylsilyl trifluoromethanesulfonate (1.1 equiv) were added to the solution of **2-2** (33 mg, 0.118 mmol) in CH_2Cl_2 at -78 ºC and stirred for 1h. The reaction was then quenched with saturated aqueous $NaHCO_3$ solution, extracted with CH_2Cl_2 and concentrated. The residue was dissolved in THF. $NaHCO_3$ (1.2 equiv) and NBS (1.2 equiv) were added sequentially at 0 ºC. The mixture was warmed to room temperature and stirred overnight. After removal of the solvents, the residue was purified with column chromatography to yield the title compound as yellow solid in 19% yield. **^{1}H NMR** (300 MHz, $CDCl_3$) δ 7.42 (d, J = 1.5 Hz, 1H), 7.23 (d, J = 1.7 Hz, 1H), 7.18 (d, J = 8.2 Hz, 1H), 4.07 (s, 2H), 3.98 (s, 2H).

***N*-(5-(2,4-Dichlorobenzyl)-1H-imidazol-2-yl)acetamide (2-4):**

A solution of **2-3** (33 mg，0.118 mmol) in DMF at 0 ºC was added the solution of N-acetylguanidine (2 equiv) in CH_2Cl_2 (1 mL). The mixture was stirred at room temperature overnight. Then water was added, and the mixture and was extracted with ethyl acetate, washed

with brine, and dried over $MgSO_4$. After filtration and concentration, the residue obtained was purified with column chromatography to yield the title compound as yellow solid in 27% yield. **^{1}H NMR** (300 MHz, $CDCl_3$) δ 7.39 (s, 1H), 7.15 (d, *J* = 8.0 Hz, 2H), 6.44 (s, 1H), 3.96 (s, 2H), 2.17 (s, 3H).

5-(2,4-Dichlorobenzyl)-1H-imidazol-2-amine (SOMCL-16-175):

A solution of **2-4** in HCl/MeOH was stirred at 60 ºC overnight. The mixture was then concentrated and the residue was purified with column chromatography to yield **SOMCL-16-175** as brown oil in 18% yield. **^{1}H NMR** (300 MHz, $CDCl_3$) δ 7.33 (s, 1H), 7.18 (t, *J* = 7.8 Hz, 2H), 6.15 (s, 1H), 4.45 – 4.19 (m, 2H), 3.83 (s, 2H); **^{13}C NMR** (126 MHz, $CDCl_3$) δ 147.62, 134.62, 133.84, 132.89, 131.49, 129.53, 127.56, 124.19, 109.33, 29.71; **ESI-MS**: 242 $[M + H]^+$; **HRMS-ESI**: *m/z* $[M + H]^+$ calcd for $C_{10}H_{10}Cl_2N_3$: 242.0252, found: 242.0246.

^{1}H, ^{13}C-NMR spectra of SOMCL-16-171 and SOMCL-16-175

^{1}H, ^{13}C-NMR spectra of SOMCL-16-171

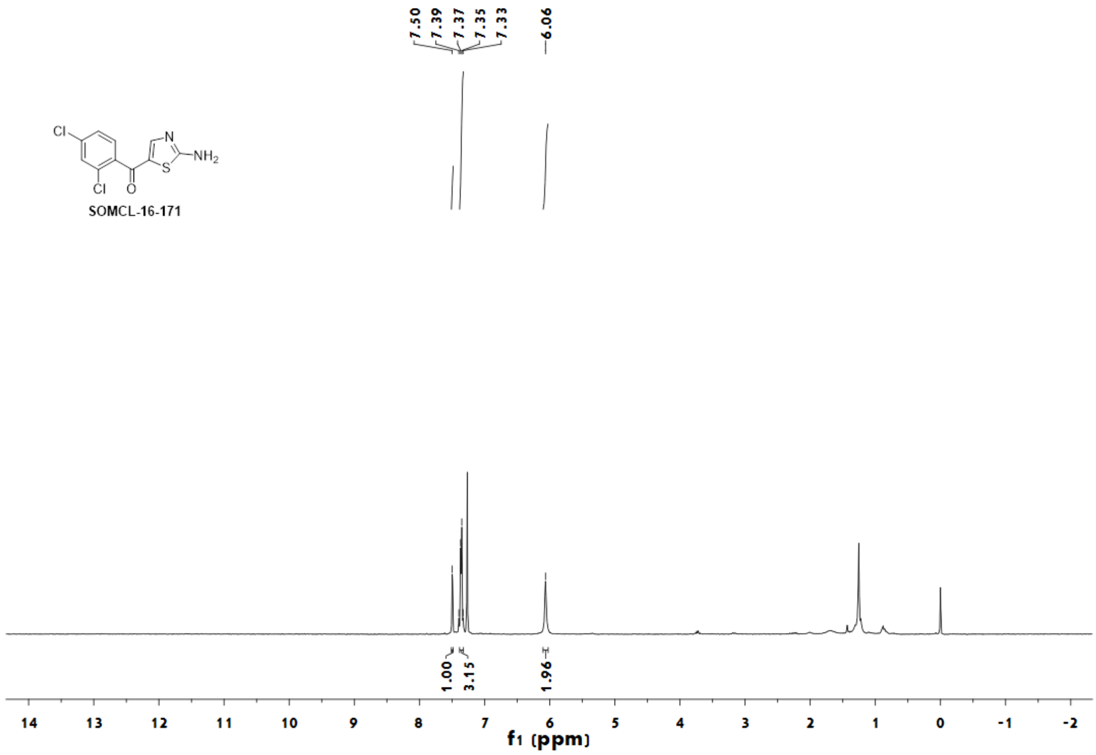

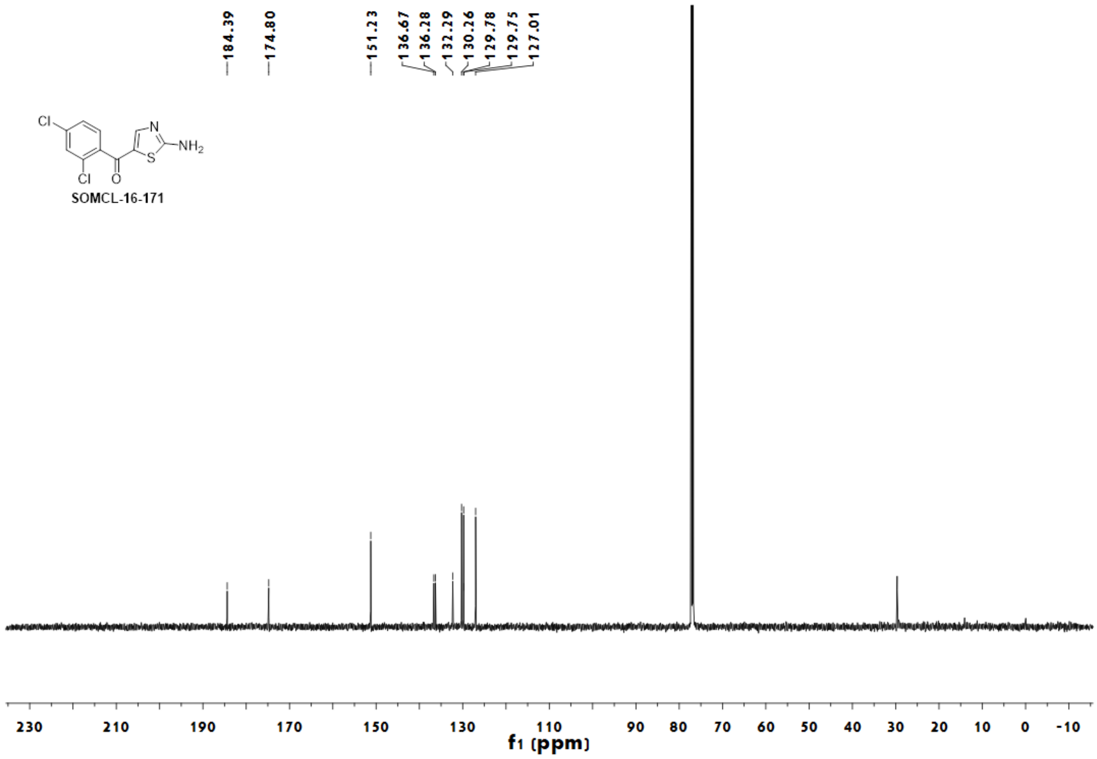

^{1}H, ^{13}C-NMR spectra of SOMCL-16-175

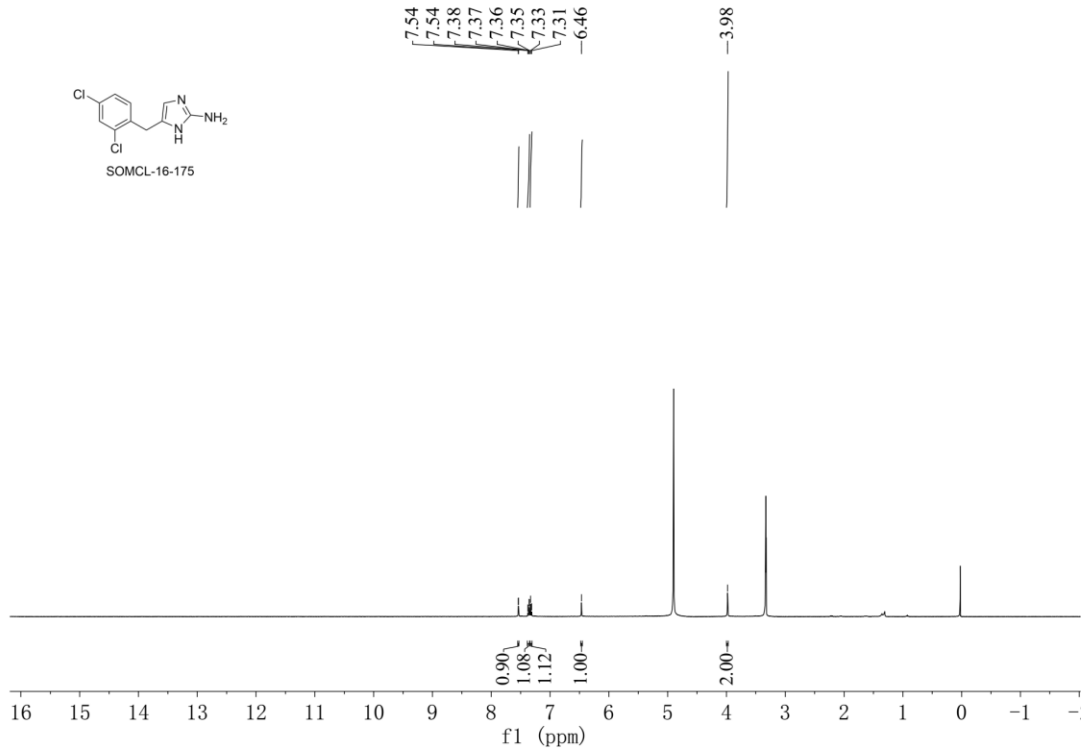

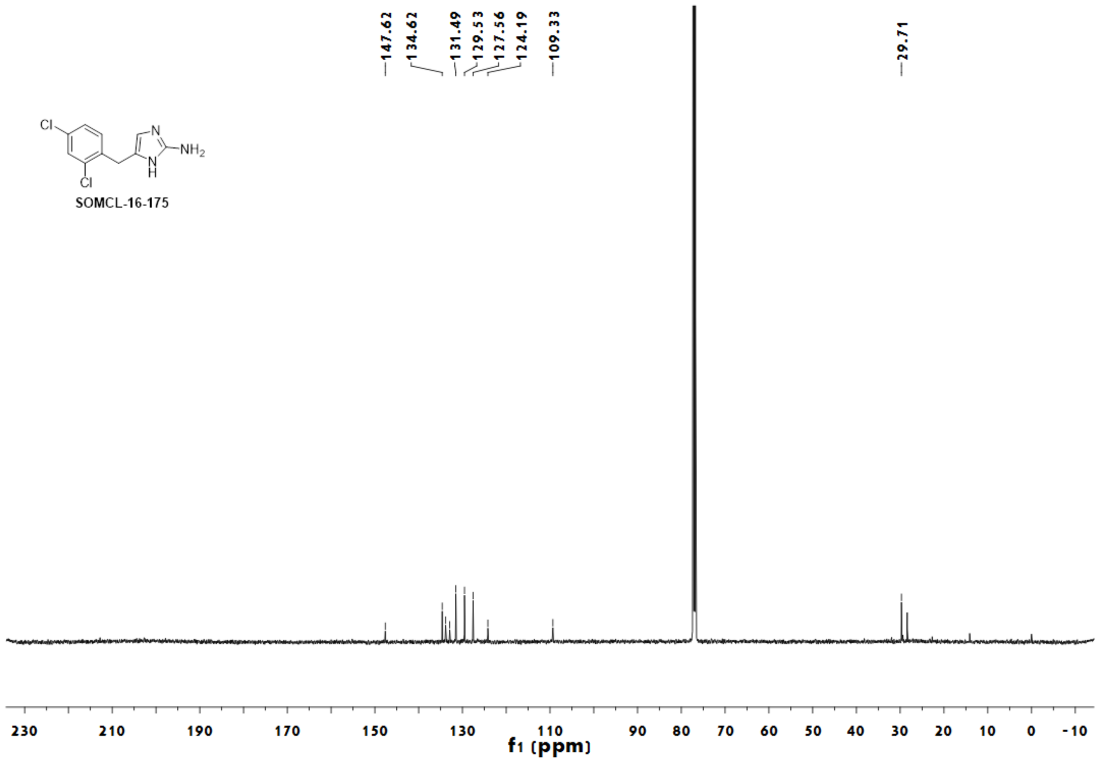

Transparent Methods

Protein sample preparation

Full-length cDNA of Hsp90α and Hsp82 were kindly provided by Dr. Liguang Lou (Shanghai Institute of Materia Medica, Chinese Academy of Sciences, China). $Hsp90^{9\text{-}236}$ (Hsp90N) plasmid was a gift from Dr. Jianhua He (National Facility for Protein Science in Shanghai, ZhangJiang Lab, China). Wide-type Sortase $A^{60\text{-}206}$ expression plasmid was a gift from Dr. Caiguang Yang (Shanghai Institute of Materia Medica, Chinese Academy of Sciences, China). $Hsp90^{293\text{-}554}$ (Hsp90M), $Hsp90^{9\text{-}240}$LPKTG, $Hsp90^{273\text{-}554}$ and $Hsp90^{9\text{-}554\Delta 241\text{-}268}$ (Hsp90 NMΔ) were sub-cloned into pET28a or pET15b vector. The mutants for $Hsp90^{293\text{-}554}$ and Sortase $A^{60\text{-}206}$ (P94S/D160N/K196T) were created by PCR.

His-tagged $Hsp90^{9\text{-}236}$, $Hsp90^{293\text{-}554}$, $Hsp90^{293\text{-}554}$ mutants, $Hsp90^{9\text{-}554\Delta 241\text{-}268}$, Hsp90, Hsp82, $Hsp90^{9\text{-}240}$LPKTG, $Hsp90^{273\text{-}554}$ and Sortase $A^{60\text{-}206}$ (P94S/D160N/K196T) were expressed in *Escherichia coli* and purified by using a combination of affinity chromatography and size exclusion chromatography on an FPLC system. $Hsp90^{273\text{-}554}$ was cleaved from its His-tag during elution from Ni-NTA resin by using thrombin. Hsp90 NMΔ with its N-terminal domain isotope labelled was prepared by following the reported protocol with a moderate modification (Freiburger et al., 2015). Hsp90 NMΔ with its N-terminal domain isotope labelled was synthesized by using two protein samples $Hsp90^{9\text{-}240}$LPKTG and $Hsp90^{273\text{-}554}$. $Hsp90^{9\text{-}240}$LPKTG and $Hsp90^{273\text{-}554}$ that were selectively labelled or unlabelled were incubated with Sortase $A^{60\text{-}206}$ (P94S/D160N/K196T) at 20 °C for 80 minutes. The molar ratio of $Hsp90^{9\text{-}240}$LPKTG:$Hsp90^{273\text{-}554}$:Sortase $A^{60\text{-}206}$ (P94S/D160N/K196T) for the protein ligation reaction is 1:2:2. To inhibit the reverse process of the ligation, protein concentrator with a molecular weight cutoff at 10 kDa was used as the container and centrifuged at 2000 × *g* during the reaction to remove the peptide byproduct. The Hsp90 NMΔ formed was subsequently purified by using a HiTrap Q HP column, followed by size exclusion chromatography. ^{15}N, ^{13}C, and ^{2}H labelled samples were produced by growth in M9 minimal media with ^{15}N labelled ammonium chloride, ^{13}C labelled glucose, and D_2O used as the nitrogen, carbon, and water sources, respectively.

NMR spectroscopy

All triple resonance experiments including HNCA, HN(CO)CA, HNCO, HN(CA)CO, HNCACB and CACB(CO)NH were recorded with ^{15}N, ^{13}C and 70% deuterium triple-labelled Hsp90$^{293-554}$ on Bruker 600 MHz NMR spectrometer equipped with a cryogenically cooled probe at 20 °C. [^{1}H, ^{15}N] HSQC spectra were acquired on Bruker 600 MHz or 900 MHz NMR spectrometers equipped with a cryogenically cooled probe at 20 °C. 1D ^{31}P spectra were acquired on Bruker 500 MHz NMR spectrometer equipped with a liquid nitrogen cooled cryoprobe at 25 °C. The spectra were processed by using NMRPipe (Delaglio et al., 1995) and analyzed with CARA (Keller, 2004) and Sparky (Kneller and Kuntz, 1993). Chemical shift perturbation values ($\Delta\delta_{avg}$) for ^{15}N and ^{1}H nuclei were derived from the following equation:

$$\Delta\delta_{avg} = \sqrt{\left((\Delta\delta_N/5)^2 + \Delta\delta_H{}^2\right)/2}$$

where $\Delta\delta_N$ and $\Delta\delta_H$ represent the observed chemical shift changes in dimension ^{15}N and ^{1}H dimension, respectively. Based on the protein concentrations and the CSP values provided by the NMR titration experiments of ^{15}N-labelled Hsp90M and **SOMCL-16-175**, dissociation constant (K_d) was determined by global fitting according to the equation below:

$$\Delta\delta_{avg} = \frac{\delta_{TOT}\left(nL_T + nP_T + K_d - \sqrt{(nL_T + nP_T + K_d)^2 - 4n^2L_TP_T}\right)}{2nP_T}$$

where $\Delta\delta_{avg}$ donates the observed chemical shift change, δ_{TOT} is the chemical shift difference between free and complexed protein, n is the binding stoichiometry, L_T is the concentration of titrant protein and P_T is the total concentration of analyte protein (Fielding, 2007).

Crystal structure determination

Crystals of Hsp90α's middle domain were obtained by using hanging drop vapor diffusion method in a solution containing 0.5 M ammonium sulfate, 29.5% (w/v) polyethylene glycol 3350 and 5% glycerol (pH 8.5) at 4 °C. The final concentration of Hsp90M used in the crystallization was 0.12 mM. The crystals obtained were cryo-protected in the crystallization buffer containing 20% (v/v) glycerol and flash cooled in liquid nitrogen. X-ray diffraction data were collected at beamline BL17U1 at the Shanghai Synchrotron Radiation Facility (Wang et al., 2018). The data were processed with HKL3000 (Minor et al., 2006). The structure was

solved by molecular replacement using the program CCP4 with a search model of PDB code 1HK7 (Bailey, 1994; Meyer et al., 2003). The models were built using coot and refined with a simulated-annealing protocol implemented in the program PHENIX (Adams et al., 2002; Emsley and Cowtan, 2004). Data collection and refinement statistics of the solved structure was shown in Table S1.

Fragment-based active compound discovery

Hit fragment compound screening with Hsp90α's middle domain as the target were carried out by following our previously reported protocol (Yu et al., 2016). In the first round of screening, 200 μM grouped fragment compound mixtures without or with the presence of 5 μM Hsp90M were dissolved in phosphate buffer (20 mM NaH_2PO_4/Na_2HPO_4, 100 mM NaCl and 2% DMSO in D_2O) and used in ligand-observed CPMG and STD spectrum acquisition. The potential hit compound candidates were then subjected to a second-round of screening by using CPMG and STD NMR experiments. 200 μM single potential hit compound without or with the presence of 5 μM Hsp90M were used in the second-round of screening. All of the ligand observed CPMG and STD NMR experiments were acquired at 25 °C on Bruker 600 MHz NMR spectrometer equipped with a cryogenically cooled probe.

General synthetic approach for ***SOMCL-16-171*** *and* ***SOMCL-16-175***

The general synthetic approach for compounds is summarized in scheme 1. The synthesis of **SOMCL-16-171** commenced from the condensation of thio-urea with DMF-DMA followed by cyclization with 2-bromo-1-(2,4-dichlorophenyl)ethanone. The synthesis of **SOMCL-16-175** was started from 2-(2,4-dichlorophenyl)acetic acid, which was first transformed to the ketone intermediates **2-2** through forming the Weinreb amide **2-1** followed by treatment with Grignard reaction. Subsequent bromination of **2-2** gave intermediate **2-3**, which was then subjected to cyclization with N-acetylguanidine and removal of acetyl under acidic condition to yield compound **SOMCL-16-175**.

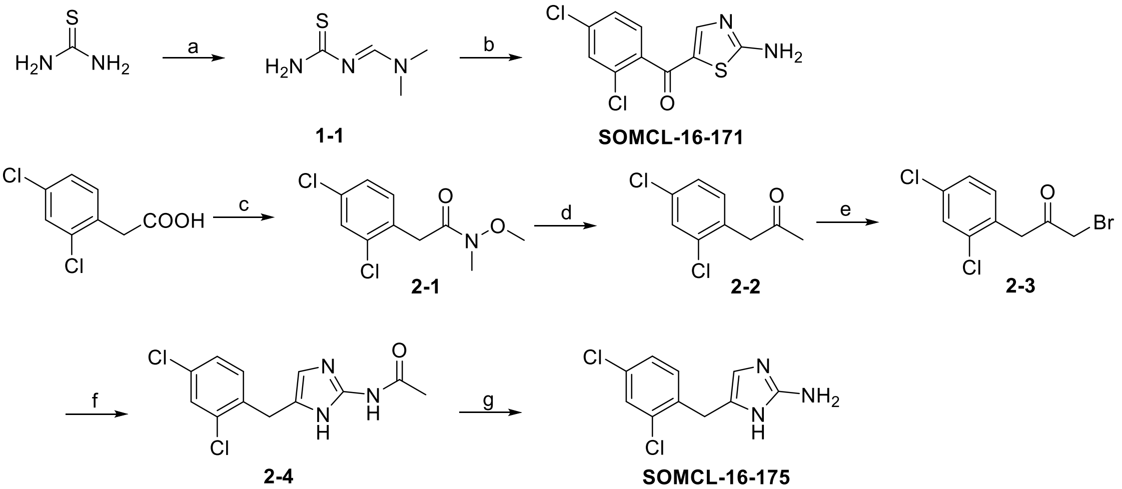

Scheme 1. Synthetic route of **SOMCL-16-171** and **SOMCL-16-175.** Reagents and conditions: (a) N,N-Dimethylformamide dimethyl acetal, MeOH, reflux, 46%; (b) 2-bromo-1-(2,4-dichlorophenyl)ethanone, Triethylamine, DCM, rt, 40%; (c) N,O-dimethylhydroxylamine for 6-22, 2,4-dichloroaniline for 6-37, HATU, HOAT, DIPEA, DCM, rt, 73-80%; (d) CH3MgBr, THF, -78℃~0℃, 77%; (e) (i) Trimethylsilyl trifluoromethanesulfonate, DIPEA, -78℃; (ii) NBS, NaHCO3, THF, 0℃~60℃, 19% (two steps); (f) N-acetylguanidine, DMF, 0℃~60℃, 27%; (g) HCl/MeOH, 60℃, 18%.

Molecular docking

The crystal structure of Hsp90α's middle domain (PDB: 6KSQ) was prepared using the Protein Preparation Wizard implemented in the Schrödinger suite (Sastry et al., 2013). This procedure added hydrogen atoms and missing side chains of residues. The orientation of polar hydrogens and the protonated states of the receptor were then optimized. The overall structure was refined using OPLS3 force field (Harder et al., 2016) with harmonic restraints on heavy atoms. The 3D structure of **SOMCL-16-175** was generated and optimized using the Ligprep tool of the Schrödinger suite, and the docking of **SOMCL-16-175** to Hsp90M was performed with the Induced Fit Docking (IFD) tool. The detailed protocol of IFD tool can be found as previously published (Koldso et al., 2010). The docking grid was centered on the centroid of eight residues: Phe349, Leu363, Asp372, Gly387, Lys 443, Glu451, Ile522, and Glu535. These residues were chosen according to the NMR studies. The following docking simulation was performed with default settings except that the extra precision (XP) mode was used in the last docking round. The final pose was selected from the top-scoring conformations.

Isothermal titration calorimetry measurements

All ITC measurements were performed at 30 °C by iTC200 calorimeter (GE Healthcare) in an ITC buffer (20 mM Tris, 75 mM NaCl, 6 mM $MgCl_2$ and 1 mM β-mercaptoethanol, pH 7.4) while stirring at 800 rpm. Hsp90 NMΔ protein samples were premixed with DMSO or either with one of two compounds (ten-fold molar excess of **SOMCL-16-171** or **SOMCL-16-175**). Pretreated protein was diluted with the ITC buffer to 50 μM, and ADP or AMPPNP was diluted with the ITC buffer to a final concentration of 1 mM. The final concentration of DMSO in the reaction buffer is 2.5% of the total volume. The titrations were performed using an initial injection of 0.4 μL followed by 19 identical injections of 2 μL ADP or AMPPNP to the cell, and last three data points were averaged to account for the heat of dilution. Data were analyzed by using the program Origin 7.0.

Thermal shift assay

Protein thermal shift experiments were performed on a 7500 fast real-time PCR system (ABI, United States). Each reaction system contains 5 × SYPRO Orange, 10 μM protein sample and 1 mM **SOMCL-16-171** or 500 μM **SOMCL-16-175** dissolved in 20 μL of thermal shift assay buffer (20 mM Tris, 75 mM NaCl and 1 mM β-mercaptoethanol, pH 7.4). The mixture samples were heated from 25 °C to 99 °C at 1% Ramp rate, and the melting curves were processed by using Protein Thermal Shift software 1.3.

ATP hydrolysis assay

The ATP hydrolysis process catalyzed by Hsp82 (Hsp90α yeast homolog) was monitored by recording ^{31}P NMR spectra on Bruker 500 MHz NMR spectrometer equipped with a liquid nitrogen cooled cryoprobe at 25 °C. The reaction systems containing 3 μM Hsp82 and 1 mM ATP without or with the presence of 500 μM **SOMCL-16-175** or 200 μM Geldanamycin (GA) were dissolved in reaction buffer (100 mM Tris, 20 mM KCl, 6 mM $MgCl_2$, pH 7.4) and incubated at 37 °C. The ^{31}P NMR spectra were acquired right after a three hour incubation.

Cellular thermal shift assay（CETSA）

Cellular thermal shift assay was conducted according to the protocol as reported (Molina et al., 2013). MDA-MB-231, MCF7 and SKBR3 cells were harvested with PBS buffer and lysed by subjecting to three freeze-thaw cycles. Then, the cell lysates were mixed and incubated

with **SOMCL-16-175** (500 μM) or **SOMCL-16-171** (1 mM) or DMSO (1 %) at room temperature for 20 minutes. After pre-incubation the mixture samples were divided into aliquots and submitted to a paralleled incubation at different temperatures ranging from 43 ℃ to 67 ℃ lasting for 5 minutes. Finally, the denatured samples were centrifuged, and the supernatants were analyzed by immunoblotting.

Cell viability and colony formation assays

Breast cancer cells MDA-MB-231, MCF7, and SKBR3 were cultured in DMEM/F12, DMEM, and RPMI 1640 medium, respectively. 10% fetal bovine serum was added into the medium during cell culturing. To test the effects of **SOMCL-16-171** and **SOMCL-16-175** on the viability of breast cancer cell lines, 4000 cells/well MDA-MB-231, 6000 cells/well MCF7, and 6000 cells/well SKBR3 were seeded to 96-well flat-bottomed microtiter plates and treated with DMSO or different concentrations of compounds (30 nM, 100 nM, 300 nM, 1 μM, 3 μM, 10 μM, 30 μM, 100 μM, 300 μM) for 72 h. The cell viability were then determined by Sulforhodamine B (S1402, Sigma) assay. The absorbance at 510 nm was measured in SpectraMax M5 (Molecular Devices), and the obtained data were analyzed by using GraphPad Prism 5.

The colony formation assays were performed using MDA-MB-231, MCF7, and SKBR3 cells with or without the treatment of **SOMCL-16-175**. 500 cells/well were plated in six-well plates and treated with either the compound (7.5 μM for MDA-MB-231 and SKBR3 cells, 17.5 μM for MCF7 cells) or 0.1% DMSO for about 7-15 days. The cell colony was fixed by using methyl alcohol, and then tinted using 0.5% crystal violet. Colony numbers were counted in Adobe Photoshop CS5.

Sample preparation and LC-MS/MS data acquisition for proteomics study

MCF7 cells were cultured and then treated by 0.1% DMSO or 35 μM **SOMCL-16-175** for 48 h. Cells were lysed with SDS lysis buffer (100-mM dithiothreitol, 4% sodium SDS, 100-mM Tris-HCl, pH 7.6). Proteins were extracted by ultrasonication (15% amplitude, 5s on and 5s off for 1 min, JY92-IIDN, Ningbo Scientz Biotechnology Co., LTD, China) and then denatured and reduced at 95°C for 5 min. Protein concentration was determined by a tryptophan-based fluorescence quantification method. The filter-aided sample preparation

(FASP) method was used for digesting proteins. Briefly, 50 μg proteins were loaded in a 10 kDa centrifugal filter tube (Millipore), washed twice with 200 μL UA buffer (8 M urea in 0.1 M Tris-HCl, pH 8.5), alkylated with 50 mM iodoacetamide in 200 μL UA buffer for 30 min in the darkness, washed thrice with 100 μL UA buffer again and finally washed thrice with 100 μL 50 mM NH_4HCO_3. All above steps were centrifuged at 12,000 g at room temperature. Proteins were digested by trypsin (1:50 of w/w, Promega Corporation, Madison, WI, USA) at 37°C for 16 h, and peptides were collected by centrifugation. Digested peptides were purified using C18 Stage-tips and evaporated to dryness in a Speed-Vac sample concentrator. Finally, ~2 μg peptides were subjected to LC-MS/MS analysis for each sample.

Peptides were separated and analyzed by coupling an Easy nano-UPLC1200 liquid chromatography (Thermo Fisher Scientific) to a Q Exactive HF mass spectrometer (Thermo Fisher Scientific). Peptides were loaded on to an in-house packed analytical column (75 μm i.d. × 30 cm, ReproSil-Pur C18-Pur, 1.9 μm, Dr. Maisch GmbH, Ammerbuch, Germany), with a 180-min gradient at a flow rate of 300 nL/min. The column was heated to 55°C using a column compartment to prevent overpressure during LC separation. Mobile phase A consisted of 0.1% formic acid, and mobile phase B consisted of 0.1% formic acid in 80% acetonitrile. The gradient was set as follows: 2%-5% B in 1 min; 5%-33% B in 145 min; 33%-45% B in 22 min; 45%-100% B in 4 min; 100% B in 8 min. The spray voltage was set at 2,300 V in positive ion mode and the ion transfer tube temperature was set at 300 °C. Data-dependent acquisition was performed using Xcalibur software in profile spectrum data type. The MS1 full scan was set at a resolution of 60,000 @ m/z 200, AGC target 3e6 and maximum IT 20 ms by orbitrap mass analyzer (350-1700 m/z), followed by 'top 20' MS2 scans generated by HCD fragmentation at a resolution of 15,000 @ m/z 200, AGC target 1e5 and maximum IT 100 ms. Isolation window was set at 1.6 m/z. The normalized collision energy (NCE) was set at NCE 27%, and the dynamic exclusion time was 40 s. Precursors with charge 1, 7, 8 and >8 were excluded for MS2 analysis.

Proteomics data analysis

All mass spectrometric data were analyzed using MaxQuant 1.6.5.0 against the human Swiss-Prot database containing 20,231 sequences (downloaded in December, 2017). Label-free

quantification (LFQ) was chosen for proteomic quantification using the default parameters. The function of "Match between runs" was used to reduce missing values in proteome quantification with a matching time window of 0.7 min and an alignment time window of 20 min. Carbamidomethyl cysteine was searched as a fixed modification. Oxidized methionine and protein N-term acetylation were set as variable modifications. Enzyme specificity was set as trypsin. The maximum missing cleavage site was set as 2. The tolerances of first search and main search for peptides were set at 20 ppm and 4.5 ppm, respectively. The minimal peptide length was set at 7. False discovery rates (FDRs) of peptide and protein were set with the cutoffs not greater than 1%.

LFQ intensity was used for proteomic data analysis. All analysis steps were conducted in Persus and R softwares. Missing values were imputed by method of normal distribution imputation in Perseus. Principal component analysis (PCA) was conducted using *factoextra* package in R. Differently expressed proteins were analyzed using a global permutation-based FDR approach implemented in Perseus. GO biological process enrichment analysis was performed using Fisher's exact test in Perseus.

Immunoblot analysis

The MCF7 cells were harvested with RIPA (P00013C, Beyotime, China) lysis buffer sitting on the ice after treated with 0.1% DMSO, 0.1 μM Geldanamycin or 35 μM **SOMCL-16-175** for 48 h. The cell debris was removed by centrifugation at 12000 rpm for 15 min, and the supernatant was collected. Protein concentrations for all of the samples submitted to western blot analysis were measured by using the Bradford method (Bradford kit, P0006C, Beyotime, China), and samples containing equal amounts of total protein were loaded for SDS-PAGE gel electrophoresis. The protein samples in polyacrylamide gel were then transferred onto 0.22 μM PVDF membrane (Millipore, United States) under a constant current running for 3 h. The membranes were blocked by using 5% defatted milk and incubated with primary antibodies (CDK1, CDK2, CDK4, Hsp70, Hsp90 and β-actin) in 4 °C for 16 h. After removing the primary antibodies, the membranes were further incubated with the secondary antibody (HRP-conjugated anti-mouse or HRP-conjugated anti-rabbit antibody) at room temperature for 2 h. Finally, chemiluminescent HRP substrate was applied to visualize specific proteins in the

membrane.

References

Adams, P.D., Grosse-Kunstleve, R.W., Hung, L.W., Ioerger, T.R., McCoy, A.J., Moriarty, N.W., Read, R.J., Sacchettini, J.C., Sauter, N.K., and Terwilliger, T.C. (2002). PHENIX: building new software for automated crystallographic structure determination. Acta Crystallogr D *58*, 1948-1954.

Bailey, S. (1994). The Ccp4 Suite - Programs for Protein Crystallography. Acta Crystallogr D *50*, 760-763.

Delaglio, F., Grzesiek, S., Vuister, G.W., Zhu, G., Pfeifer, J., and Bax, A. (1995). NMRPipe: a multidimensional spectral processing system based on UNIX pipes. Journal of biomolecular NMR *6*, 277-293.

Emsley, P., and Cowtan, K. (2004). Coot: model-building tools for molecular graphics. Acta Crystallogr D *60*, 2126-2132.

Fielding, L. (2007). NMR methods for the determination of protein-ligand dissociation constants. Prog Nucl Mag Res Sp *51*, 219-242.

Freiburger, L., Sonntag, M., Hennig, J., Li, J., Zou, P.J., and Sattler, M. (2015). Efficient segmental isotope labeling of multi-domain proteins using Sortase A. Journal of biomolecular NMR *63*, 1-8.

Harder, E., Damm, W., Maple, J., Wu, C.J., Reboul, M., Xiang, J.Y., Wang, L.L., Lupyan, D., Dahlgren, M.K., Knight, J.L*., et al.* (2016). OPLS3: A Force Field Providing Broad Coverage of Drug-like Small Molecules and Proteins. J Chem Theory Comput *12*, 281-296.

Keller, R.L.J. (2004). The Computer Aided Resonance Assignment Tutorial. Cantina Verlag.

Kneller, D.G., and Kuntz, I.D. (1993). Ucsf Sparky - an Nmr Display, Annotation And Assignment Tool. J Cell Biochem, 254-254.

Koldso, H., Severinsen, K., Tran, T.T., Celik, L., Jensen, H.H., Wiborg, O., Schiott, B., and Sinning, S. (2010). The Two Enantiomers of Citalopram Bind to the Human Serotonin Transporter in Reversed Orientations. J Am Chem Soc *132*, 1311-1322.

Meyer, P., Prodromou, C., Hu, B., Vaughan, C., Roe, S.M., Panaretou, B., Piper, P.W., and Pearl, L.H. (2003). Structural and functional analysis of the middle segment of Hsp90: Implications for ATP hydrolysis and client protein and cochaperone interactions. Molecular cell *11*, 647-658.

Minor, W., Cymborowski, M., Otwinowski, Z., and Chruszcz, M. (2006). HKL-3000: the integration of data reduction and structure solution--from diffraction images to an initial model

in minutes. Acta crystallographica Section D, Biological crystallography *62*, 859-866.

Molina, D.M., Jafari, R., Ignatushchenko, M., Seki, T., Larsson, E.A., Dan, C., Sreekumar, L., Cao, Y.H., and Nordlund, P. (2013). Monitoring Drug Target Engagement in Cells and Tissues Using the Cellular Thermal Shift Assay. Science *341*, 84-87.

Sastry, G.M., Adzhigirey, M., Day, T., Annabhimoju, R., and Sherman, W. (2013). Protein and ligand preparation: parameters, protocols, and influence on virtual screening enrichments. J Comput Aid Mol Des *27*, 221-234.

Wang, Q.S., Zhang, K.H., Cui, Y., Wang, Z.J., Pan, Q.Y., Liu, K., Sun, B., Zhou, H., Li, M.J., Xu, Q*., et al.* (2018). Upgrade of macromolecular crystallography beamline BL17U1 at SSRF. Nucl Sci Tech *29*.

Yu, J.L., Chen, T.T., Zhou, C., Lian, F.L., Tang, X.L., Wen, Y., Shen, J.K., Xu, Y.C., Xiong, B., and Zhang, N.X. (2016). NMR-based platform for fragment-based lead discovery used in screening BRD4-targeted compounds. Acta pharmacologica Sinica *37*, 984-993.
